# BRUTUS-LIKE (BTSL) E3 ligase-mediated fine-tuning of Fe regulation negatively affects Zn tolerance of Arabidopsis

**DOI:** 10.1093/jxb/erad243

**Published:** 2023-07-02

**Authors:** Camilla Stanton, Jorge Rodríguez-Celma, Ute Krämer, Dale Sanders, Janneke Balk

**Affiliations:** Department of Biochemistry and Metabolism, John Innes Centre, Norwich NR4 7UH, UK; Department of Biochemistry and Metabolism, John Innes Centre, Norwich NR4 7UH, UK; Faculty of Biology and Biotechnology, Ruhr University Bochum, D-44801 Bochum, Germany; Department of Biochemistry and Metabolism, John Innes Centre, Norwich NR4 7UH, UK; Department of Biochemistry and Metabolism, John Innes Centre, Norwich NR4 7UH, UK; School of Biological Sciences, University of East Anglia, Norwich NR4 7TJ, UK; MPI of Molecular Plant Physiology, Germany

**Keywords:** Arabidopsis, BRUTUS, iron deficiency, transcriptomics, ubiquitin ligase, zinc

## Abstract

The mineral micronutrients zinc (Zn) and iron (Fe) are essential for plant growth and human nutrition, but interactions between the homeostatic networks of these two elements are not fully understood. Here we show that loss of function of *BTSL1* and *BTSL2*, which encode partially redundant E3 ubiquitin ligases that negatively regulate Fe uptake, confers tolerance to Zn excess in *Arabidopsis thaliana*. Double *btsl1 btsl2* mutant seedlings grown on high Zn medium accumulated similar amounts of Zn in roots and shoots to the wild type, but suppressed the accumulation of excess Fe in roots. RNA-sequencing analysis showed that roots of mutant seedlings had relatively higher expression of genes involved in Fe uptake (*IRT1*, *FRO2*, and *NAS*) and in Zn storage (*MTP3* and *ZIF1*). Surprisingly, mutant shoots did not show the transcriptional Fe deficiency response which is normally induced by Zn excess. Split-root experiments suggested that within roots the BTSL proteins act locally and downstream of systemic Fe deficiency signals. Together, our data show that constitutive low-level induction of the Fe deficiency response protects *btsl1 btsl2* mutants from Zn toxicity. We propose that BTSL protein function is disadvantageous in situations of external Zn and Fe imbalances, and formulate a general model for Zn–Fe interactions in plants.

## Teaser

Mutation of two E3 ligases that suppress iron uptake in roots also confers tolerance to zinc toxicity, identifying a regulatory point of interaction between iron and zinc homeostasis.

## Introduction

Zinc (Zn) and iron (Fe) are two important elements in biology, with plants serving as the primary source of these micronutrients for human nutrition. However, population groups relying upon diets of mainly cereals, which are a poor source of bioavailable Zn and Fe (Cakmak *et al.*, 2010), are likely to suffer deficiencies. It is estimated that at least 2 billion people globally are affected, with Fe and Zn deficiency often occurring simultaneously in individuals, leading to a range of health disorders, including anaemia, impaired development, and depressed immune system function, which can lead to early childhood morbidity (WHO, 2010).

Zn and Fe are found in the *d-*block of the periodic table, alongside other transition metals, such as manganese (Mn) and copper (Cu). Zn and Fe have different chemical characteristics, with Zn found as a stable divalent cation (Zn^2+^), whilst Fe can take on different oxidation states (Fe^2+^ and Fe^3+^) and participate in oxidation–reduction reactions (Habashi, 2013a, b). As such, the ions fulfil very different biochemical roles in enzymes and proteins. However, the divalent cations of Fe^2+^, Zn^2+^, and Mn^2+^ have similar ionic radii and favour similar coordination geometries, which can lead to protein mismetallation (Robinson and Glasfeld, 2020). Moreover, these metals can share the same membrane transporters. A particularly well-studied example in plants is the Iron-Regulated Transporter1 (IRT1), the main uptake route of Fe (as Fe^2+^) in the root epidermis, which is also able to transport Zn^2+^, Mn^2+^, Co^2+^, Cd^2+^, and Ni^2+^ (Eide *et al.*, 1996; Vert *et al.*, 2002; Nishida *et al.*, 2011).

The question of how plants coordinate the uptake and distribution of different transition metals in relation to one another has been studied in some detail for Fe and Zn (Hanikenne *et al.*, 2021). Physiological studies as well as Arabidopsis mutant studies have provided important insights into the points of crosstalk in the homeostatic networks of these two metals (summarized in Table 1), but many questions remain. An early observation is that excess Zn in the soil or medium results in secondary Fe deficiency and Fe deficiency-related phenotypes, such as chlorosis, growth stunting (Vert *et al.*, 2002; Fukao *et al.*, 2011; Leskova *et al.*, 2017), and induction of the transcriptional response to Fe deficiency (Fukao *et al.*, 2011; Leskova *et al.*, 2017). Increasing the Fe concentration in the medium ameliorates Zn toxicity (Shanmugam *et al.*, 2011). These observations could in part be explained by post-translational regulation of IRT1, which helps prevent the uptake of non-Fe metals: a cytosolic loop of IRT1 binds directly to Zn or Mn ions, which leads to internalization of IRT1 from the plasma membrane and subsequent degradation (Barberon *et al.*, 2014; Dubeaux *et al.*, 2018). Thus, when Zn levels in the environment are high, excess uptake of Zn into the plant would be prevented by active degradation of IRT1, but this also decreases Fe import. While this mechanism may be effective at moderate Zn levels, at higher concentrations seedlings do accumulate Zn and display toxicity symptoms (Fukao *et al.*, 2011; Leskova *et al.*, 2017). In addition, an elevated Fe concentration suppresses Zn toxicity because their cations compete for binding sites on a number of other membrane transporters and chelators during the acquisition of these metals and their movement within the plant.

**Table 1.** Genes and metabolites affecting Zn tolerance in Arabidopsis

Manipulation	More/less Zn tolerant	Direct effect	Effect on metal distribution
Increased Fe supply	More	Increased competition between Fe and Zn for uptake and transport	Increased shoot Fe accumulation and reduced root Zn accumulation (Shanmugam *et al.*, 2011)
*fbp* (mutation of *FBP*, *FIT*-*BINDING PROTEIN*)	More	Increased transcriptional activity of FIT, including de-repression of *NAS1/2/4* in the root stele	Increased retention of Zn in roots and Fe translocation to shoots (Chen *et al.*, 2018)
RNAi of *MTP3* (*METAL TOLERANCE PROTEIN 3*)	Less	Reduced expression of *MTP3*, a vacuole-localized importer of Zn in the roots	Reduced retention of Zn in root vacuoles and increased shoot Zn accumulation (Arrivault *et al.*, 2006)
*35S:MTP3*	More	Ectopic expression of the Zn vacuolar importer *MTP3* in rosette leaves	Enhanced Zn accumulation in shoot vacuoles (Arrivault *et al.*, 2006)
*HMA3* ^*Col-0*^ (*HEAVY METAL ATPASE*, Col-0 accession)	Less	Non-functional variant of vacuolar Zn importer *HMA3*	Reduced Zn retention in root vacuoles (Morel *et al.*, 2009; Park and Ahn, 2017)
*nas4-1* (mutation of *NICOTIANAMINE SYNTHASE 4*)	Less	Reduced NA levels in roots and shoots	Lower capacity of Fe uptake and root-to-shoot transport (Palmer *et al.*, 2013)
*35S:ZIF1* (*ZINC-INDUCED FACILITATOR 1*)	More	High, ectopic expression of vacuolar NA importer *ZIF1*	Increased NA transport into root vacuoles leading to increased Zn sequestration in roots (Haydon *et al.*, 2012)
*zif2* (mutation of *ZINC-INDUCED FACILITATOR 2*)	Less	Reduced expression of vacuolar NA importer *ZIF2*	Decreased NA transport into root vacuoles, leading to reduced Zn sequestration in roots and enhanced translocation to the shoot (Remy *et al.*, 2014)
*gsh1* (mutation of *GLUTATHIONE SYNTHASE 1*)	Less	Reduced glutathione biosynthesis	Reduced Fe levels in shoots (Shanmugam *et al.*, 2012)
*frd3* (mutation of *FERRIC REDUCTASE DEFECTIVE 3*)	More	Reduced efflux of citrate into the xylem as a result of loss of function of the citrate transporter FRD3	20 μM Zn (moderate excess) in the medium rescues root growth and oxidative stress in the *frd3* mutant by competing with Fe in the root cell wall (Scheepers *et al.*, 2020)
*FRD3* ^*Sha*^ (Shahdara accession)	More	Mild impairment of FRD3 function leading to reduced efflux of citrate into the xylem	Reduced Fe (and Zn) translocation to shoots (Pineau *et al.*, 2012)
*bts-1* (T-DNA insertion in the 5ʹUTR of *BRUTUS*)	More	Reduced expression of *BTS*, encoding an E3 ubiquitin ligase which targets IVc bHLH transcription factors for degradation	Up-regulation of Fe uptake and transport genes, increased Fe accumulation in roots and shoots, increasing tissue Fe/Zn ratio (Zhu *et al.*, 2022)
*myb10 myb72* (mutation of Myb transcription factors *MYB10* and *MYB72*)	Less	Reduced expression of *NAS4* and other Fe uptake and transport genes controlled by *MYB10* and *MYB72*	Decreased Fe uptake capacity (Palmer *et al.*, 2013)

Another important point of crosstalk between Fe and Zn is the FER-like Iron deficiency induced Transcription factor FIT (Hanikenne *et al.*, 2021). Functioning as a heterodimer together with basic helix–loop–helix (bHLH) 38/39/100/101 (group Ib) proteins, FIT up-regulates genes involved in Fe uptake and translocation such as *IRT1*; genes involved in vacuolar Zn transport; and *Nicotianamine Synthase* (*NAS*) genes required for both processes (Schwarz and Bauer, 2020; Fan *et al.*, 2021). Several studies have documented the specific roles of those FIT-regulated genes in Zn homeostasis or Fe–Zn crosstalk (see Table 1). In addition, regulators of FIT activity such as FIT-binding protein (FBP) and the E3 ligase BRUTUS (BTS) have recently been shown to influence Zn toxicity phenotypes (summarized in more detail below).

Of the vacuolar Zn transporters regulated by FIT, the *MTP3* gene encoding Metal Tolerance Protein 3 is expressed in root epidermal and cortical cells (Arrivault *et al.*, 2006). *MTP3* RNAi lines accumulated higher levels of Zn in shoot tissues under Zn excess and Fe deficiency compared with control lines (Arrivault *et al.*, 2006). As such, immobilization of Zn into root vacuoles by MTP3 decreases Zn mobility in the root and thus limits its root to shoot translocation. FIT also up-regulates *Heavy Metal ATPase3* (*HMA3*), encoding a vacuolar Zn/Cd/Co/Pb transporter found to be expressed in the root stele and shoots (Morel *et al.*, 2009; Wu *et al.*, 2012). The *Arabidopsis thaliana* Col-0 accession carries a loss-of-function *HMA3* allele, correlated with greater sensitivity to Zn excess due to reduced Zn sequestration in roots (Park and Ahn, 2017).

The FIT-regulated *NAS* genes have a more complex role in Zn and Fe transport and distribution. They are up-regulated under Zn excess and Fe deficiency, as well as Zn deficiency (van de Mortel *et al.*, 2006; Klatte *et al.*, 2009; Chen *et al.*, 2018). Nicotianamine (NA) is able to form stable complexes with a range of metals, including Zn^2+^, Fe^2+^, and Mn^2+^, and mediates radial and long-distance metal transport, intracellular transport, and vacuolar storage (Scholz *et al.*, 1992; Bauer and Schuler, 2011). In addition, T-DNA *nas4* mutants showed reduced NA levels in both roots and shoots and were more sensitive to Fe deficiency, displaying interveinal chlorosis (Koen *et al.*, 2013), as well as more sensitivity to excess Zn (Palmer *et al.*, 2013). NA additionally plays a role in promoting root Zn sequestration in roots. The Zinc-Induced Facilitator 1 (ZIF1) and 2 (ZIF2) proteins are tonoplast-localized NA transporters that mediate vacuolar localization of NA-chelated metals in conjunction with MTP3 and HMA3 (von Wiren *et al.*, 1999; Haydon and Cobbett, 2007; Haydon *et al.*, 2012; Remy *et al.*, 2015).

In addition to NA, citrate and glutathione (GSH) are also important chelators shared by Zn and Fe, as well as Mn, for inter- and intracellular transport (Morrissey and Guerinot, 2009; Shanmugam *et al.*, 2012; Flis *et al.*, 2016). Fe is transported to shoots via the xylem predominantly as Fe^3+^–citrate complexes, and there is also evidence for the presence of Zn–citrate complexes in xylem sap (White, 1981; Lopez-Millan *et al.*, 2000; Rellán-Álvarez *et al.*, 2011; Cornu *et al.*, 2015; Flis *et al.*, 2016). Citrate efflux into the xylem is mediated by Ferric Reductase Defective 3 (FRD3), which is induced under Fe deficiency and Zn excess, as well as Zn deficiency (Durrett *et al.*, 2007; Pineau *et al.*, 2012; Charlier *et al.*, 2015). *frd3* mutants showed impaired root-to-shoot Fe transport and constitutive activation of the Fe deficiency response, resulting in very strong accumulation of various secondary substrates of IRT1, in particular Zn and Mn, in shoots (Delhaize, 1996; Baxter *et al.*, 2008), as well as accumulation of Fe in cell walls of the vasculature in roots (Rogers and Guerinot, 2002; Durrett *et al.*, 2007). Supply of moderate amounts of Zn (20 µM) partially restored root growth by interfering with the accumulation of Fe and hydrogen peroxide in the cell wall (Scheepers *et al.*, 2020). The role of GSH in metal homeostasis is still poorly understood, and may be mediated by its direct Fe- and metal-binding ability, or its role in scavenging reactive oxygen species in the ascorbate–glutathione cycle, or in nitric oxide signalling upstream of the Fe deficiency response (Xiang *et al.*, 2001; Shanmugam *et al.*, 2015; Zlobin *et al.*, 2017). Loss-of-function *gsh1* mutants, which have reduced GSH levels, show greater sensitivity to Zn excess and are unable to launch an Fe-mediated Zn tolerance phenotype due to diminished root-to-shoot Fe translocation (Shanmugam *et al.*, 2012).

While the role of different transporters and chelators in Fe and Zn homeostasis is fairly well characterized, the role of regulatory proteins is only just emerging. The recently discovered FBP inhibits the activity of FIT in the root stele by binding to the DNA-binding domain (Chen *et al.*, 2018). *fbp* mutants show increased expression of *NAS* genes and tolerance to Zn excess. FIT protein levels are also regulated post-transcriptionally by the Fe-binding E3 ubiquitin ligases, BRUTUS-LIKE1 (BTSL1) and BTSL2. The *btsl1 btsl2* double mutant fails to down-regulate the Fe deficiency response upon Fe resupply, correlating with sustained levels and activity of FIT (Rodriguez-Celma *et al.*, 2019). The closely related BTS protein negatively regulates the transcriptional response to Fe deficiency upstream of FIT (Long *et al.*, 2010; Selote *et al.*, 2015; Hindt *et al.*, 2017). Expressed in shoots and the root stele, BTS can interact with IVc bHLH transcription factors bHLH104, ILR3, and bHLH115 (Long *et al.*, 2010; Selote *et al.*, 2015; Kim *et al.*, 2019), as well as possibly bHLH121 (Kim *et al.*, 2019), and thus facilitate their degradation (Lichtblau *et al.*, 2022). RNA-seq analysis of *bts* mutant seedlings showed constitutive up-regulation of Fe uptake and transport genes (Zhu *et al.*, 2022), enhanced Fe accumulation, and tolerance to Fe deficiency (Hindt *et al.*, 2017), but the *bts-1* mutant also showed tolerance to Zn excess (Zhu *et al.*, 2022). In contrast, the role of BTSL1 and BTSL2 in Zn homeostasis has yet to be explored.

Here we show that *btsl1 btsl2* double mutant seedlings display tolerance to Zn excess (100 µM), partially modulated by the Fe concentration in the medium. RNA-sequencing (RNA-seq) analysis, ionomics, and split-root experiments suggest that root-specific up-regulation of the ferrome, via both FIT-dependent and -independent networks, and moderately increased Fe uptake, mitigates the Zn-induced Fe deficiency response in the mutant. We propose that tight regulation of FIT by the E3 ubiquitin ligases BTSL1 and BTSL2 under conditions of mild Fe deficiency may have a trade-off in that the homeostatic networks fail when Fe and Zn levels in the soil are very unbalanced.

## Materials and methods

### Plant materials and growth conditions

*Arabidopsis thaliana* Columbia ecotype (Col-0) plants were used as wild type. T-DNA insertion lines *btsl1-1* (SALK_015054), *btsl2-2* (SALK_048470), and the *btsl1-1 btsl2-2* double mutant (*btsl1x2*) were previously described by Rodriguez-Celma *et al.* (2019).

Plants were surface sterilized and grown on 0.25× modified Hoagland medium with 1% (w/v) sucrose and 1.5% (w/v) EDTA-washed agar as described by Sinclair and Krämer (2020). The medium was composed of (in mM): Ca(NO_3_)_2_ (1.5), KH_2_PO_4_ (0.28), MgSO_4_ (0.75), KNO_3_ (1.25), CuSO_4_ (0.005), ZnSO_4_ (0.001 or 0.1), MnSO_4_ (0.005), H_3_BO_3_ (0.025), Na_2_MoO_4_ (0.0001), and KCl (0.05). Fe was added in the form of Fe(NO_3_)_3_HBED [*N,N*ʹ-bis(2-hydroxyphenyl)ethylenediamine-*N,N*ʹ-diacetic acid], prepared by suspending 10.5 mM HBED (Santa Cruz Biotechnology Inc., TX, USA) in 10 mM Fe(NO_3_)_3_ solution and added to a concentration of 0.005, 0.05, or 0.1 mM as indicated. The pH was adjusted to 5.7 with 1 mM MES. Seedlings were grown vertically on 100 mm^2^ square plates (R & L Slaughter) in a controlled-environment room maintained at 23 °C with a photoperiod of 16 h light/8 h dark. For propagation and soil experiments, plants were grown on a peat mix; 600 litres of Levington F2 peat, 100 litres of 4 mm grit, 196 g of Exemptor® (chloronicotinyl insecticide, GB84080896A, Bayer CropScience, UK) under 22 °C with a photoperiod of 16 h light/8 h dark. For growth on soil with excess Zn, soil was dried overnight at 45 °C in a drying oven and subsequently watered and mixed with a ZnSO_4_ solution to reach final concentration of 1000 mg kgDW^–1^.

### Growth of plants with split-root systems

Seedlings were germinated on 0.25× Murashige and Skoog medium with 1% (w/v) agar (AGA03, Formedium) and 0.75% (w/v) sucrose buffered to pH 5.7. To generate split roots, at 5 d old the primary root was excised and seedlings grown for an additional 5 d. Seedlings with two main roots of similar length were transferred to 100 mm^2^ round split plates (Gosselin™, 15438784, Fisher Scientific, UK) containing modified Hoagland medium with either 1 µM ZnSO_4_, 100 µM ZnSO_4_, or 0 µM FeHBED.

### Chlorophyll quantification

Chlorophyll was extracted from dried and powdered 14-day-old seedlings (~50 mg). Samples were incubated in 2 ml of 100% (v/v) methanol at room temperature with shaking (350 rpm) for 10 min and then centrifuged for 5 min at 10 000 *g*. A 1 ml aliquot of solution was transferred to a transparent cuvette on ice and absorbance measured at 650 nm and 665 nm using a spectrophotometer to calculate the total chlorophyll concentration according to (Lichtenthaler, 1987): Chl *a*+*b* (μg ml^–1^)=(22.5×*A*_650_)+(4×*A*_665_).

### Inductively coupled plasma-optical emission spectrometry (ICP-OES)

For elemental analysis, root and shoot samples were harvested separately (~200 mg). Apoplastic cations were desorbed from roots by washing samples in ice-cold 2 mM CaSO_4_ and 10 mM EDTA for 10 min followed by 0.3 mM bathophenanthroline disulfonate and 5.7 mM sodium dithionite for 3 min as described by Cailliatte *et al.* (2010). Samples were washed twice with dH_2_O then dried for 48 h at 65 °C and the dry weight recorded. Dried samples were then digested in 2 ml of 65% (v/v) HNO_3_ and 0.5 ml of H_2_O_2_ for 4 h at 95 °C. The digested solution was diluted to 15 ml with ultrapure water and analysed by ICP-OES (Vista-PRO CCD Simultaneous ICP-OES; Agilent Technologies) calibrated with standards: Zn and Fe at 0.2, 0.4, 0.6, 0.8, and 1 mg l^–1^ and Mn at 1, 2, 3, 4, and 5 mg l^–1^. Soft winter wheat flour was used as a reference material (RM 8438; U.S. National Institute of Standards and Technology) and analysed in parallel with all experimental samples.

### Ferric chelate reductase (FCR) activity

Roots (~200 mg) from 14-day-old seedlings were excised, transferred to a 2 ml Eppendorf tube, and submerged in 700 µl of assay solution (0.1 mM Fe^3+^-EDTA and 0.3 mM ferrozine). Samples were incubated in the dark for 30 min at room temperature. The solution was then transferred to a cuvette and absorbance at 562 nm was measured using a spectrophotometer (Emre Aksoy and Koiwa, 2013). Root surface FCR activity (μmol Fe^2+^) gFW^–1^ h^–1^ was calculated assuming a molar extinction coefficient of 28.6 mM^–1^ cm^–1^ according to the formula {[(*A*_562_/28.6)×700]/root FW}×2.

### Quantitative reverse transcription–PCR (qRT–PCR)

Total RNA was extracted from 100 mg of frozen and powdered leaf or root tissue sampled from pooled 14-day-old seedlings using the RNeasy Plant Mini Kit (74904, QIAGEN). RNA was then extracted as per the manufacturer’s instructions, with a final elution volume of 30 µl, followed by DNase treatment (Turbo DNase kit, Agilent). cDNA synthesis was carried out in 20 µl final volume reactions with 200–500 ng of mRNA using Invitrogen™ M-MLV reverse transcriptase (28025013, Thermo Fisher Scientific). qRT–PCR was carried out in 20 µl reactions using 20 ng of cDNA and SYBR® Green JumpStart™ ReadyMix™ (S4438, Sigma-Aldrich). *ACTIN2* and *TIP41* were used as reference genes and relative gene expression was determined using the 2^–∆∆Ct^ method (Livak and Schmittgen, 2001).

### RNA-sequencing

RNA was extracted from separated shoots and roots of 14-day-old seedlings using an RNeasy Plant Mini Kit (74904, QIAGEN). Quality check was carried out on a 2100 Bioanalyzer Instrument (G2939BA, Agilent Technologies) using the Agilent RNA 6000 Nano Kit. Samples were diluted to 100 ng µl^–1^, and a total volume of 30 µl was sent for sequencing. Construction of cDNA sequencing libraries and next-generation sequencing (NGS) were carried out by Novogene (Hong Kong). Libraries were generated using an NEBNext® Ultra™ RNA Library Prep Kit for Illumina (NEB) and then sequencing was run on an Illumina Novaseq 6000 (Illumina, CA, USA) using 150 bp paired-end reads. Quality control was carried out on raw reads using FastQC (Andrew, 2010).

### Read alignment and differential gene expression (DGE)

Reads were pseudoaligned to the TAIR10 transcriptome (TAIR10_cdna_20101214_updated.fa) and transcript abundance calculated using Kallisto (v 0.46.1) (Bray *et al.*, 2016) on the NBI high-performance computing (HPC) platform (NBI, Norwich, UK). DGE analysis was then performed using Sleuth (Pimentel *et al.*, 2017) with a 2-fold (log_2_FC >1) cut-off and a 5% false discovery rate (adjusted *P*-value, *q*-value <0.05) using pairwise comparisons between samples. Sleuth was also used to run principal component analysis (PCA). Kallisto and Sleuth were both run in R Studio. Results were verified by performing DGE analysis with EdgeR on the Degust platform (Powell, 2015), and the top results were compared.

### Bioinformatic analysis of RNA-seq data

Shoot and root data were analysed separately. Gene list comparisons and Venn diagrams were generated using Gene List Comparator (Cermak, 2020). Hierarchical clustering of differentially expressed genes based on FC difference from the mean was performed using Morpheus (https://software.broadinstitute.org/morpheus) based on Pearson correlation and the average linkage method, as well as generation of heatmaps. Gene Ontology (GO) (Mi *et al.*, 2019) for biological processes, and Kyoto Encyclopedia of Genes and Genomes (KEGG) pathway (Kanehisa and Goto, 2000; Kanehisa, 2019; Kanehisa *et al.*, 2019) enrichment analysis were performed using David (Database for Annotation, Visualization and Integrated Discover) (Huang da *et al.*, 2009a, b) filtered with an adjusted *P*-value (Benjamini <0.05).

## Results

### *btsl1 btsl2* double mutant seedlings are tolerant to excess Zn

Previous work showed that the *btsl1 btsl2* double knockout mutant (further called *btsl1x2*) is more tolerant to Fe deficiency and more sensitive to Fe excess than wild-type seedlings, correlating with constitutively induced Fe uptake in the mutant (Hindt *et al.*, 2017; Rodriguez-Celma *et al.*, 2019). *btsl1x2* mutants also accumulated more Zn but not more Mn (Hindt *et al.*, 2017); however, growth in response to these divalent metal ions has not been investigated.

**Fig. 1.**
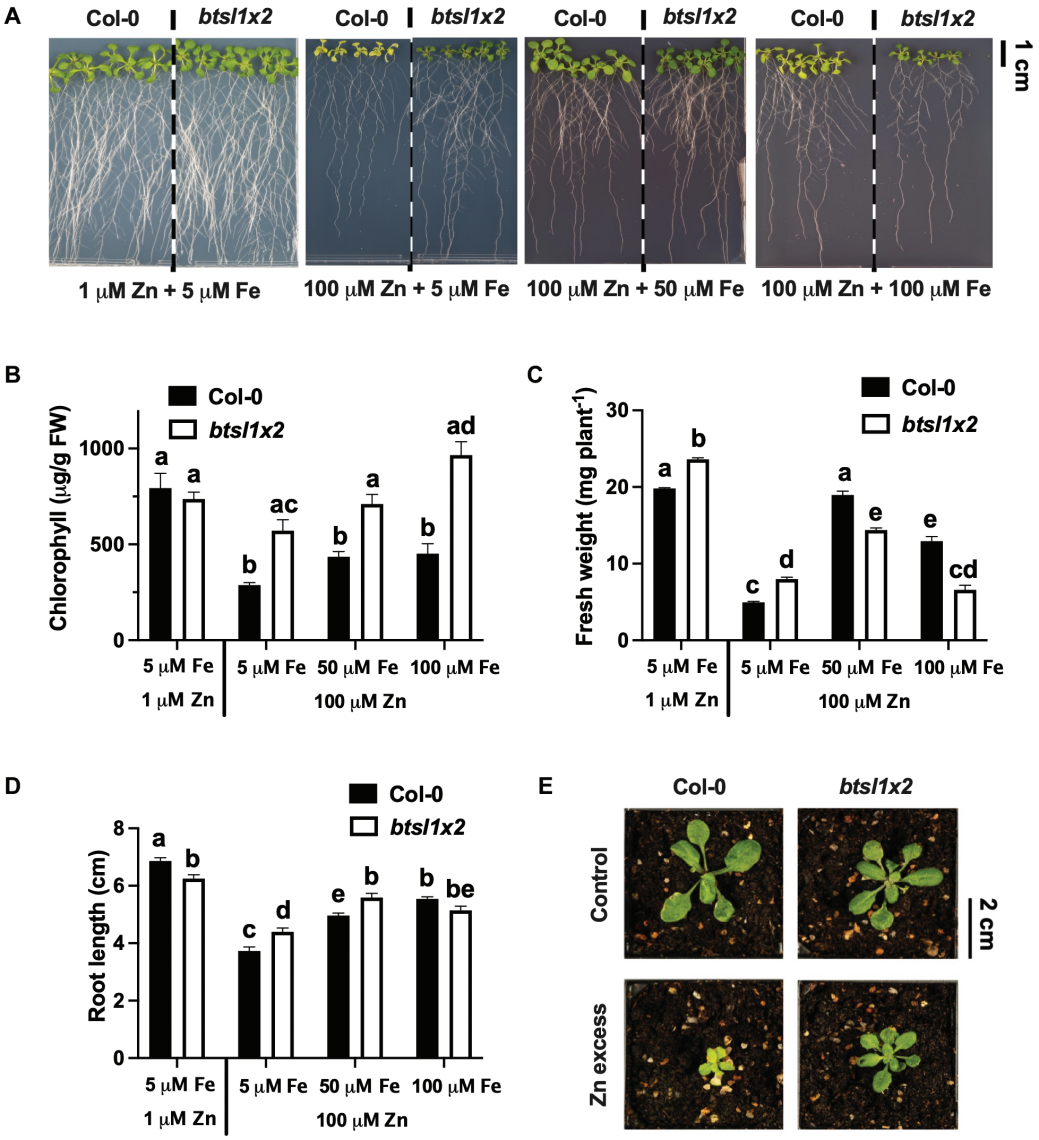
The *btsl1 btsl2* double mutant displays Zn tolerance, partially modulated by Fe in the medium. (A) Representative photographs of 14-day-old seedlings of the wild type (Col-0) and *btsl1 btsl2* double mutant (*btsl1x2*), grown on modified Hoagland medium with 1 μM ZnSO_4_ (standard conditions) or 100 μM ZnSO_4_ (excess), in the presence of 5, 50, or 100 μM FeHBED as indicated. (B) Total chlorophyll normalized to FW in shoots from seedlings in (A). (C) Shoot biomass, in mg FW, of seedlings in (A). (D) Primary root length of 10-day-old seedlings. (E) Representative photographs of Col-0 and *btsl1x2* plants grown for 22 d on soil without (Control) and with added ZnSO4 (Zn excess) at a concentration of 1 g kg^–1^ DW. For (B–D), data represent mean values (±SEM) from three independent experiments, each comprising six plants per genotype and condition. Statistically significant differences are indicated by letters (*P*<0.05) as determined by two-way ANOVA followed by Tukey HSD post-hoc test. Non-significant differences are not indicated.

First, we compared growth phenotypes of the *btsl* single mutants and the *btsl1x2* double mutant on medium with a standard level of Zn (1 μM ZnSO_4_) or with Zn excess (100 μM ZnSO_4_), containing the same concentration of Fe (5 µM FeHBED). The toxic effect of Zn on Arabidopsis seedlings manifested itself in chlorosis and decreased biomass (Fig. 1) as previously shown (Vert *et al.*, 2002; Fukao *et al.*, 2011; Leskova *et al.*, 2017). In 14-day-old wild-type seedlings grown on excess Zn, the levels of chlorophyll were 30% compared with seedlings grown on standard medium (Fig. 1B). In contrast, the *btsl1x2* mutant retained 2-fold more chlorophyll than the wild type in the presence of 100 µM Zn (570 ± 57 μg chlorophyll gFW^–1^ versus 300 ± 13 μg chlorophyll gFW^–1^, *P* < 0.01, Fig. 1B). Both the *BTSL1* and *BTSL2* genes contribute to Zn-induced chlorosis, as no significant increase in chlorophyll was observed in the single mutants (Supplementary Fig. S1B). Increasing the Fe concentration to 50 µM or 100 µM, known to suppress the effects of Zn toxicity in wild-type seedlings (Shanmugam *et al.*, 2011), increased chlorophyll levels in the wild type and restored chlorophyll levels in *btsl1x2* to an even greater degree (Fig. 1B).

Shoot biomass was higher in *btsl1x2* than in the wild type exposed to 1 µM or 100 µM Zn (120% and 160%, respectively), but lower than in the wild type at higher Fe concentrations (Fig. 1C). Root growth was inhibited in the *btsl1x2* mutant on standard medium, most probably due to toxic effects of Fe accumulation (Hindt *et al.*, 2017; Rodriguez-Celma *et al.*, 2019). Under Zn excess, *btsl1x2* seedlings grew longer roots than the wild type in the presence of 5 µM and 50 µM Fe, but not with 100 µM Fe (Fig. 1D). The higher chlorophyll concentration and shoot biomass were also evident in the *btsl1x2* mutant when grown on soil with excess Zn (1000 mg ZnSO_4_ kg^–1^ soil) (Fig. 1E).

Zn and Fe homeostasis has also been shown to be interlinked with the homeostasis of other divalent cations, such as Mn^2+^ (Rai *et al.*, 2021). To test the growth response of the *btsl1x2* mutant to Mn, seedlings were grown on increasing MnSO_4_ concentrations ranging from Mn deficiency (0 μM MnSO_4_) to excess (250 μM MnSO_4_; Supplementary Fig. S2A). There were no significant differences in chlorophyll levels between genotypes or Mn concentrations (Supplementary Fig. S2B). The *btsl1x2* mutant had more shoot biomass on 0 μM Mn but less biomass on 50 µM and 250 μM Mn compared with the wild type (Supplementary Fig. S2C), and shorter root lengths on 5 µM and 50 μM Mn (Supplementary Fig. S2D). Thus, the *btsl1x2* mutant was more tolerant to Mn deficiency but less tolerant to excess, similar to its growth response to Fe.

Together, these data show that loss of both *BTSL1* and *BTSL2* gene expression results in enhanced tolerance to Zn excess, with further suppression of toxicity effects when higher concentrations of Fe are supplied to the medium, until this also impairs growth.

### *btsl1x2* seedlings under Zn toxicity accumulate similar amounts of Zn but much less Fe in roots than the wild type

**Fig. 2.**
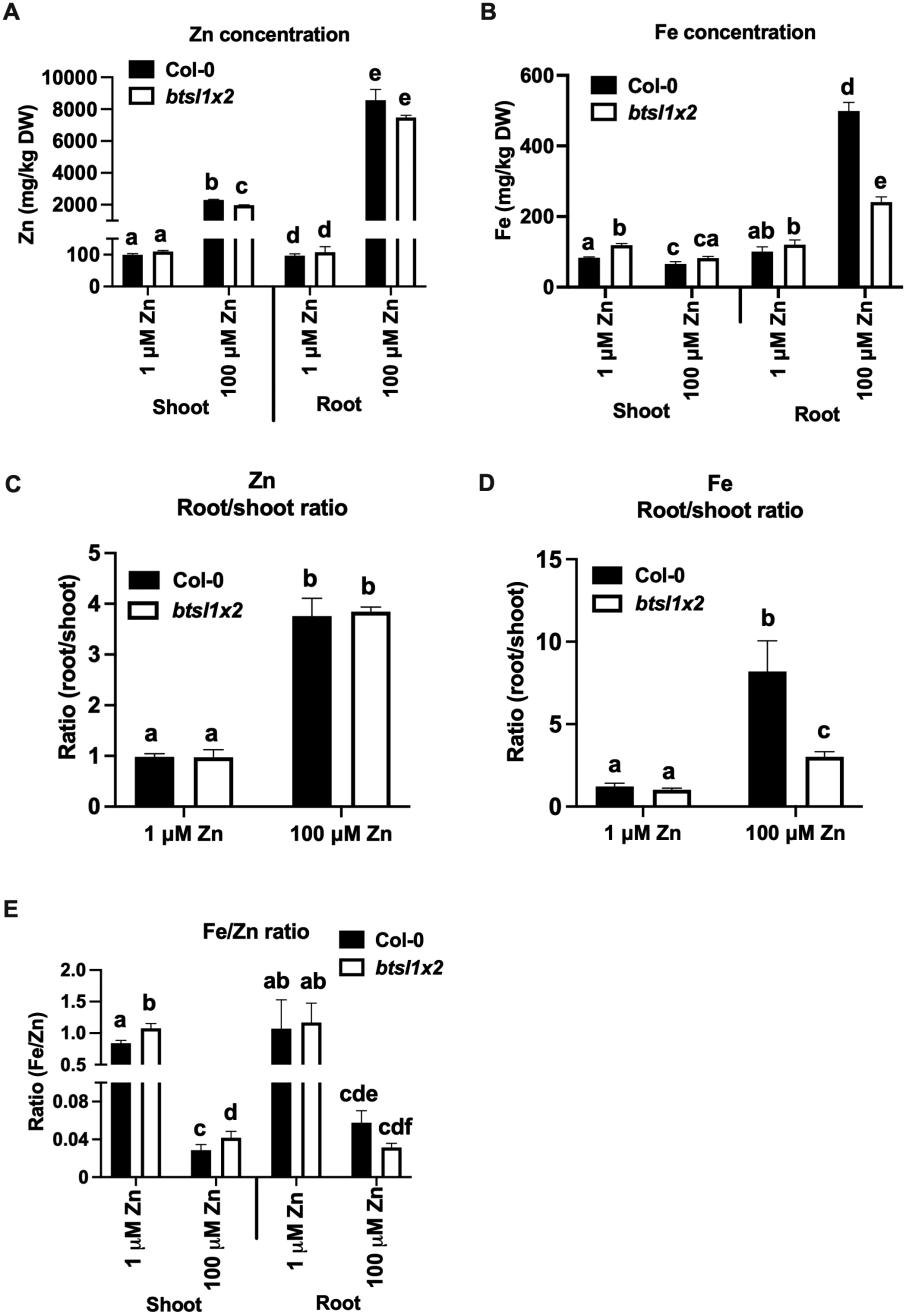
Distribution of Fe and Zn between roots and shoots in response to excess Zn. The concentration of (A) Zn and (B) Fe in the roots and shoots of 14-day-old seedlings of the wild type (Col-0) and *btsl1 btsl2* double mutant (*btsl1x2*) grown on standard (1 μM ZnSO_4_, 5 μM FeHBED) or Zn excess (100 μM ZnSO_4_, 5 μM FeHBED). Roots were desorbed with chelators to remove apoplastic cations prior to element analysis. Data represent mean values (±SEM) from three independent experiments, each comprising 10 plants per genotype and condition. Statistically significant differences are indicated by letters (*P*<0.05) as determined by two-way ANOVA followed by Tukey HSD post-hoc test. (C) Root-to-shoot ratio of Zn and (D) Fe.

To explore whether the BTSL proteins affect the distribution of Zn, Fe, or Mn between roots and shoots, elemental analysis was carried out by ICP-OES on 14-day-old seedlings grown on medium with standard or excess Zn. Roots were washed with EDTA to ensure desorption of apoplastic cations. There was no significant difference in the shoot or root Zn concentrations between the wild type and *btsl1x2* under standard growth conditions (Fig. 2A). As expected, the Zn concentration in both shoots and roots increased dramatically in wild-type seedlings exposed to 100 µM ZnSO_4_ (23-fold and 89-fold, respectively) and by a similar amount in the *btsl1x2* mutant (17-fold and 69-fold, respectively).

Consistent with previous reports (Hindt *et al.*, 2017; Rodriguez-Celma *et al.*, 2019), *btsl1x2* seedlings accumulated 1.4-fold more Fe in shoots compared with the wild type when grown under standard conditions (120 ± 5 mg Fe kg^–1^ versus 80 ± 2 mg Fe kg^–1^, *P*<0.001), whereas the roots contained similar Fe concentrations to the wild type (Fig. 2B). Induction of the Fe deficiency response by excess Zn led to 5-fold Fe accumulation in wild-type roots, and very little of this Fe was translocated to shoots. The *btsl1x2* mutant also accumulated Fe in the roots when grown under Zn excess, but only about half as much as wild-type roots (240 ± 15 mg Fe kg^–1^ versus 500 ± 24 mg Fe kg^–1^, *P*<0.01, Fig. 2B). The calculated root/shoot ratio of Zn was similar in the wild type and *btsl1x2*; however, the Fe root/shoot ratio was 8 in the wild type and 3 in *btsl1x2* (Fig. 2C, D). A reduced accumulation of Fe in roots and a lower Fe root/shoot ratio was previously observed in the *fbp* mutant under Zn excess (Chen *et al.*, 2018). Conversely, the *bts-1* mutant showed increased root Fe accumulation under Zn excess compared with the wild type (Zhu *et al.*, 2022). In terms of the Fe/Zn ratio, this was higher in the shoots of *btsl1x2* compared with wild-type seedlings (1.08 versus 0.84, Fig. 2E) and, together with the higher chlorophyll levels and shoot biomass in the mutant, indicates that the relatively small trend towards a more physiological Fe and Zn balance may suppress the Zn toxicity effects when Fe is the limiting nutrient (Fig. 1). In contrast, the root Fe/Zn ratio was relatively lower in the *btsl1x2* mutant (0.06 in the wild type versus 0.32 in *btsl1x2*, Fig. 2E) while root length was increased compared with the wild type (Fig. 1D).

Under standard growth conditions, there was no significant difference in Mn shoot or root accumulation between the *btsl1x2* mutant and wild type (Supplementary Fig. S2E). Under Zn excess, the Mn concentrations were reduced in both genotypes, particularly in the roots, with only minor differences between the genotypes. The Mn root/shoot ratio was reduced in both genotypes under Zn excess, but there was no difference between genotypes (Supplementary Fig. S2F).

In summary, the most striking difference in metal distribution in high Zn conditions is that *btsl1x2* seedlings exhibit suppressed Fe accumulation in the roots, with only minor effects on Zn, Fe, or Mn concentrations in the shoots.

### Distinct transcriptomic changes are observed in *btsl1x2* roots, but not shoots, in response to Zn excess

**Fig. 3.**
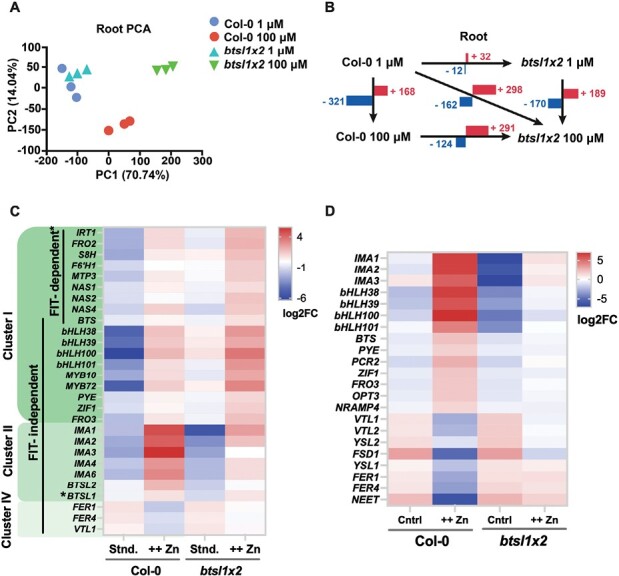
The *btsl1 btsl2* double mutant has a distinct transcriptomic response to Zn excess. RNA-seq analysis of root samples of wild-type (Col-0) and *btsl1 btsl2* (*btsl1x2*) double mutant seedlings, grown for 14 d on standard medium (1 µM ZnSO_4_, 5 μM FeHBED) or with excess Zn (100 µM ZnSO_4_, 5 μM FeHBED). (A) Principal component analysis (PCA) of root sample transcripts: PC1, principal component 1; PC2, principal component 2; (%), the percentage of variance explained by each PC. (B) Number of significantly [adjusted *P*-value >0.05, log2 (fold change) ≥1] up-regulated (+) or down-regulated (–) genes between all pairwise comparisons. (C) Heatmap of significantly differentially expressed genes [2-fold (log2FC >1) cut-off and adjusted *P*-value, *q*-value <0.05] selected from a core set of Fe deficiency-responsive genes as defined in Mai *et al.* (2016), in roots and (D) shoots. Expression is represented as the SD from the mean global gene expression of pooled replicates. Hierarchical clustering of all differentially expressed genes is shown in Supplementary Figs S5 and S8.

To investigate changes in gene expression that could underpin Zn tolerance in the *btsl1x2* mutant, RNA-seq analysis was carried out separately on roots and shoots of seedlings grown on standard (1 μM ZnSO_4_) and high Zn (100 μM ZnSO_4_) medium (Fig. 3; Supplementary Fig. S3). PCA of the RNA-seq data showed good reproducibility of the data for the three biological replicates of each genotype and treatment (Fig. 3A; Supplementary Fig. S3A). The gene expression in wild-type and *btsl1x2* roots clustered closely together under standard conditions but diverged when exposed to excess Zn (Fig. 3A).

Consistent with close clustering of samples, compared with the wild type, only 44 genes were differentially expressed in *btsl1x2* roots under standard conditions (32 up-regulated, 12 down-regulated, Fig. 3B). Of this gene set, 28 of those up-regulated (including *bHLH38*, *bHLH39*, *bHLH100*, *bHLH101*, *MYB10*, *MYB72*, and *FRO3*) and nine of those down-regulated (including abiotic stress-responsive *CYB7B122* and flowering time regulator *FLC*) were also differentially expressed in response to Zn excess (Fig. 3C; Supplementary Dataset S1), suggesting that a number of Zn excess-associated genes are constitutively more highly expressed in *btsl1x2.*

Comparing the mutant and wild type under Zn excess, 415 genes were differentially expressed in roots (291 up-regulated, 124 down-regulated, Fig. 3B) and 771 genes in shoots (207 up-regulated and 564 down-regulated, Supplementary Fig. S3B). Under Zn excess compared with standard conditions, 489 genes were differentially expressed in wild-type roots, and 359 in *btsl1x2* roots (Fig. 3B). In contrast, only 29 genes were differentially expressed in the shoots of the *btsl1x2* mutant in response to excess Zn, whereas wild-type shoots had 1277 differentially expressed genes (Supplementary Fig. S3B; Fig. 3D). This suggests that mutation of the *BTSL1* and *BTSL2* genes, which are expressed in the roots, shields the shoots from the toxic effects of excess Zn in the medium.

### *btsl1x2* roots have induced expression of Fe homeostasis and Zn storage genes under standard conditions and excess Zn, but lower expression of *IMA* genes

GO enrichment analysis showed that in roots, genes associated with Zn and Fe homeostasis were significantly up-regulated in both genotypes under Zn excess, whilst commonly down-regulated genes were enriched for terms associated with oxidative and biotic stress (Supplementary Fig. S4A). Genes that were uniquely up-regulated in the *btsl1x2* mutant under both standard conditions and Zn excess are associated with Fe homeostasis and transcriptional regulation, in agreement with the demonstrated role of BTSL2 in regulating activity of the FIT transcription factor (Rodriguez-Celma *et al.*, 2019).

To further explore how gene expression was impacted in the *btsl1x2* mutant, hierarchical clustering of all the significant Differentially Expressed Genes (DEGs) in roots was performed (Supplementary Fig. S5) and the expression pattern of a well-documented core set of genes involved in Fe and Zn homeostasis (van de Mortel *et al.*, 2006; Mai *et al.*, 2016; Gao *et al.*, 2019) was investigated (Fig. 3C). Cluster I contained genes that were strongly up-regulated in both genotypes under Zn excess but were expressed more highly in the mutant than in the wild type under both standard and Zn excess conditions. FIT-regulated genes, such as *IRT1*, *FRO2*, *MTP3*, and *NAS1/2/4*, were found in this cluster, consistent with the accumulation of FIT protein in the *btsl1x2* mutant (Rodriguez-Celma *et al.*, 2019). Interestingly, FIT-independent genes were also found in this cluster, including FIT-interacting partners *Ib bHLH* genes (*bHLH38/29/100/101*), *MYB10/72*, *PYE*, *FRO3*, and the Zn tolerance-associated *ZIF1* (Fig. 3C), in support of the suggestion that other bHLH transcription factors may be targeted by BTSL1 and BTSL2 (Lichtblau *et al.*, 2022). Increased expression of Fe homeostasis genes, including *IRT1*, *Ib bHLH* genes, and *FRO2*, has also been observed in the *bts-1* mutant under Zn excess compared with the wild type, although the genes were not up-regulated under standard conditions (Zhu *et al.*, 2022). Moreover, increased expression of *NAS* genes was previously shown to underlie the Zn tolerance phenotype of the *fbp* mutant, although other Fe deficiency-responsive genes were down-regulated in *fbp* (Chen *et al.*, 2018) that are not down-regulated in *btsl1x2*.

Cluster II contained genes that showed generally lower levels of expression in the *btsl1x2* mutant under standard conditions, and were also up-regulated in response to Zn excess in both genotypes, but to a lesser degree in the mutant than in the wild type (Fig. 3C). Fe deficiency-induced *IMA* signalling peptides were found in this cluster. The phloem-mobile *IMA* signalling peptides are thought to induce Fe deficiency transcriptional responses in the roots by inhibiting BTS and BTSL protein interactions with IVb and IVc bHLH transcription factors (Li *et al.*, 2021; Lichtblau *et al.*, 2022). Reduced expression of *IMA* genes in *btsl1x2* is consistent with the reduced Zn toxicity symptoms and transcriptomic response in shoots (Supplementary Figs S4, S6). As expected, the expression of *BTSL1* and *BTSL2* was also reduced in the mutant, serving as an internal control for the dataset. Cluster IV contained genes that were down-regulated in both genotypes in response to Zn excess but were more Zn responsive in the wild type than in the mutant (Fig. 3C). Fe storage ferritin genes *FER1* and *FER4*, and the vacuolar Fe transporter, *VTL1*, were found in this cluster.

To verify some of the RNA-seq data, qRT–PCR analysis was performed of selected genes *bHLH38* and *bHLH39* (Supplementary Fig. S7A, B), *ZIF1* and *FRO3* (Supplementary Fig. S7C, D), and *IRT1* and *FRO2* (Supplementary Fig. S7E, F) in root samples. The expression of the six genes was enhanced in the *btsl1x2* mutant under Zn excess, in agreement with RNA-seq data.

In shoots, genes uniquely up-regulated in the wild type in response to Zn were associated with oxidative and biotic stress, whilst down-regulated genes were involved in photosynthesis (Fig. 3D; Supplementary Fig. S6) in agreement with growth impairment and chlorosis (Fig. 1). Furthermore, major Fe homeostasis genes, such as *Ib bHLH* genes, *BTS*, and *PYE* were strongly up-regulated in the wild type, but not in the mutant (Fig. 3D; Supplementary Fig. S8). This confirms that wild-type shoots respond to Zn as if they are physiologically Fe deficient, but surprisingly *btsl1x2* shoots do not have this response. However, *IMA* transcripts encoding signalling peptides were partially up-regulated in mutant shoots, and *VTL1/2* transporters were partially down-regulated, in response to Zn (Fig. 3D; Supplementary Fig. S8). While this could indicate that some iron deficiency is sensed in *btsl1x2* shoots, for example in a specific tissue or cell type, it is also possible that the BTSL–FIT module affects these genes indirectly.

Together, transcriptomic analysis shows that BTSL1 and BTSL2 negatively regulate the expression of Fe homeostasis and Zn tolerance-associated genes of both FIT-dependent and -independent networks. Under Zn excess, the transcript levels of genes for Fe uptake, NA biosynthesis, and Zn storage are induced in the *btsl1x2* mutant roots, but not in shoots, except for *IMA* paralogues.

### Ferric chelate reductase activity is suppressed under Zn excess in wild-type roots compared with *btsl1x2* roots

**Fig. 4.**
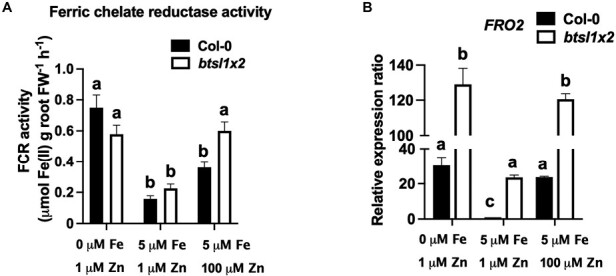
The *btsl1 btsl2* double mutant has higher FCR activity than the wild type under Zn excess. (A) Ferric chelate reductase (FCR) activity and (B) transcript levels of *FRO2*, the gene responsible for FCR activity on the root surface. Wild-type (Col-0) and *btsl1 btsl2* mutant (*btsl1x2*) seedlings were grown on medium lacking Fe (0 μM FeHBED, 1 μM ZnSO_4_), standard conditions (5 μM FeHBED, 1 μM ZnSO_4_), or excess Zn (5 μM FeHBED, 100 μM ZnSO_4_) for 10 d, after which roots were excised for the activity assays and qRT–PCR. Fold change is relative to Col-0 and normalized to reference genes *ACTIN2* and *TIP41*. Data represent mean values (±SEM) from three independent experiments, using five seedlings per measurement. Statistically significant differences are indicated by letters (*P*<0.05) as determined by two-way ANOVA followed by Tukey HSD post-hoc test.

Whilst increased expression of Fe uptake genes in the *btsl1x2* mutant is consistent with increased Fe accumulation in the shoots under standard growth conditions, it does not explain why Fe accumulation in the roots is suppressed under Zn excess (Fig. 2B). To see if Fe uptake capacity in the mutant was impacted post-transcriptionally under Zn excess, FCR activity was measured and compared with *FRO2* transcript levels. In wild-type roots, FCR activity is induced under both Fe deficiency and Zn excess, but the magnitude of induction is 2-fold lower under Zn excess compared with Fe deficiency (Fig. 4A) despite comparable transcript levels (Fig. 4B). In the *btsl1x2* mutant, FCR activity is similar in roots grown under Fe deficiency or Zn excess, and thus 2-fold higher than in Zn-exposed wild-type roots. Counterintuitively, wild-type roots accumulate more Fe per dry weight than *btsl1x2* roots under Zn excess (Fig. 2), despite lower FCR activity than *btsl1x2* under these conditions (Fig. 4A). A possible explanation for these findings is that in wild-type seedlings, FCR activity mediated by FRO2 is suppressed post-transcriptionally due to excess Zn. Subsequently, an apoplastic pool of Fe accumulates, which is not removed by EDTA washes during metal measurements and possibly is similar to the Fe accumulation in cortex cells observed in *irt1* mutants (Quintana *et al*., 2022). In the *btsl1x2* mutant, Fe that is internalized can be transported to the shoots, and indeed a slight increase in Fe is seen in shoots (Fig. 2B) which is more significant when taking the larger shoot mass into account (Fig. 1B).

### BTSL proteins act locally in Zn-exposed or Fe-deficient roots

**Fig. 5.**
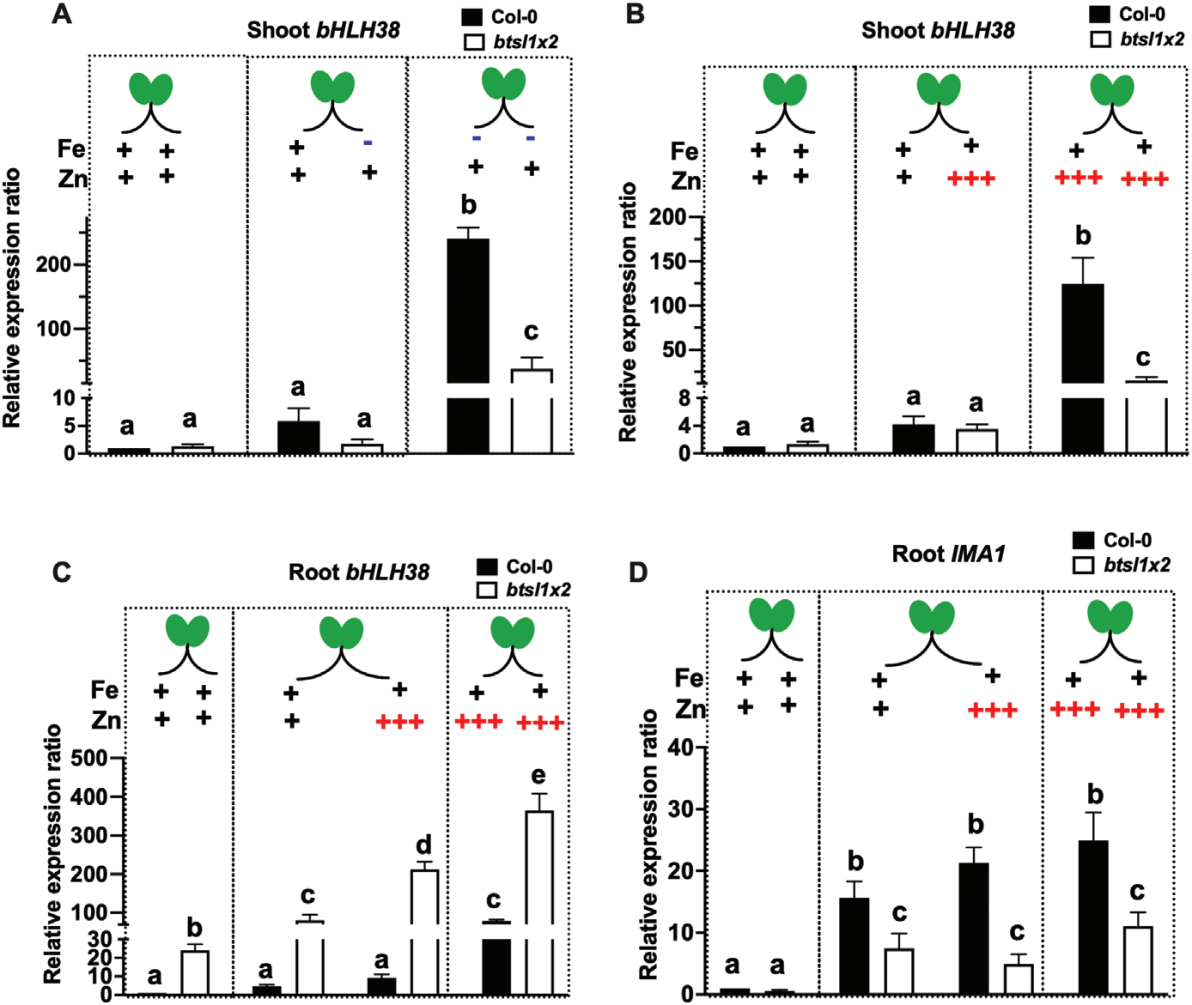
Systemic Fe signalling in *btsl1 btsl2* mutant seedlings in response to Fe deficiency and Zn excess. Wild-type (Col-0) and *btsl1 btsl2* mutant (*btsl1x2*) seedlings were germinated on standard medium (1 μM ZnSO_4_, 5 μM FeHBED). The primary root was excised at day 5 to promote development of lateral roots. At 10 d, seedlings were transferred to split agar plates with the indicated Fe and Zn concentrations. Samples for RNA extraction were taken 7 d later. (A, B) Expression of *bHLH38* in shoots in response to the roots being exposed to (A) Fe deficiency or (B) Zn excess. (C, D) Expression of (C) *bHLH38* and (D) *IMA1* in roots in response to Zn excess exposure in different halves of the root system. Fold change is relative to Col-0 control and normalized to reference genes *ACTIN2* and *TIP41*. Statistical analysis as in Fig. 4B.

To investigate the contribution of both local and systemic signals to the Zn-induced transcriptional response, we performed split-root experiments. Similar experiments were instrumental in demonstrating that apoplastic Fe causes local induction of Fe uptake genes as well as a shoot-to-root signal acting systemically on the root transcriptional response (Vert *et al.*, 2003), later confirmed by many other studies. As a marker of Zn-induced changes in both shoots and roots, the differentially expressed, FIT-independent, *bHLH38* gene was selected based on our RNA-seq analysis (Fig. 3C) and quantified by qRT–PCR. First, we verified the effect of exposing half of the root system to Fe deficiency on *bHLH38* expression in the shoots, as this has not been reported in the literature to our knowledge, but the information would be useful for comparison with the Zn excess response. Seedlings were germinated on standard medium for 10 d, then transferred to split agar plates, with one half of the root system exposed to standard conditions (5 μM FeHBED) and the other half to Fe deficiency (0 μM) for another 7 d (Fig. 5A). When only half the root system was exposed to Fe deficiency, there was no significant difference between the wild type and *btsl1x2* in terms of chlorophyll content or root length (Supplementary Fig. S9A, B). However, when the entire root system was exposed to Fe deficiency, *btsl1x2* retained significantly higher chlorophyll levels than the wild type (Supplementary Fig. S9A). This situation resulted in only a slight increase in *bHLH38* transcript levels in the shoots in both the wild type and *btsl1x2* (6- and 2-fold, respectively), compared with a dramatic 300-fold increase in *bHLH38* expression when the whole root system of wild-type seedlings is Fe deficient. A similar expression pattern was observed for bHLH39 in the shoot (Supplementary Fig. S9C). Thus, the availability of Fe to one half of the root system prevents a transcriptional Fe deficiency response in the shoots.

When half the root system was exposed to Zn (Fig. 5B), the expression of *bHLH38* was similarly not highly induced in the shoots, or in Zn-exposed roots of the wild type. In contrast, *bHLH38* was increasingly induced in *btsl1x2* roots, with a significant difference between the Zn-exposed half versus the non-exposed half of the root system (Fig. 5C, 210- versus 80-fold, *P*>0.01). The same phenomenon was seen for the *IRT1* transcript (Supplementary Fig. S10D) and when half the *btsl1x2* roots were exposed to Fe deficiency (Supplementary Fig. S10C, D). Thus, as derepression of Fe deficiency-responsive genes is stronger in the root half that is exposed to excess Zn in *btls1x2*, this suggests local action by BTSL1/2.

Recently, IMA1 has been implicated in the shoot-to-root Fe signalling pathway (Grillet *et al.*, 2018; García *et al.*, 2022) and suggested to interact with BTSL proteins (Lichtblau *et al.*, 2022). Consistent with this, and with its appearance in Cluster II of the RNA-seq analysis (Fig. 3C), *IMA1* transcript levels increase similarly in both halves of the root, the one maintained in standard conditions and the one exposed to excess Zn, in the wild type (15- and 21-fold, respectively), and also in *btsl1x2* (7-fold and 4-fold, respectively, Fig. 5D). In contrast, a gene which is in the transcriptional module of BTSL1/2, such as *bHLH38* (Fig. 5C), is more highly expressed in root halves of *btsl1x2* exposed to excess Zn than under standard conditions. This can be taken to further support that IMA1 function is positioned upstream of BTSL. We attribute the overall quantitative differences between genotypes under both conditions to the difference in Zn tolerance, with *btsl1x2* having a lower degree of physiological Fe deficiency caused by excess. Similarly, there was no significant difference in the level of *FER1* induction between standard and Zn-exposed roots in either genotype (Supplementary Fig. S10B), as *FER1* is not BTSL regulated. In summary, the split-root data suggest that BTSL proteins act downstream of systemic Fe deficiency signals.

## Discussion

**Fig. 6.**
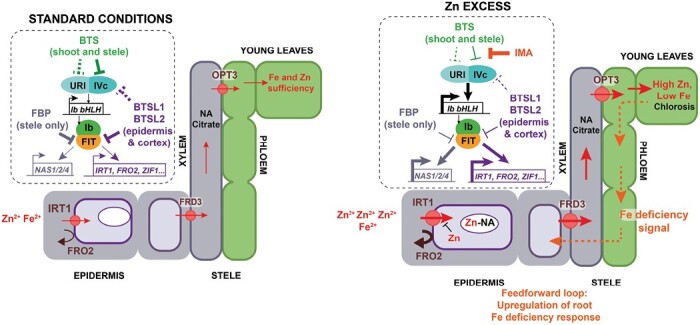
Model for Arabidopsis response to Zn excess. Under standard growth conditions (Fe/Zn ratios of ~5–25), Fe uptake is controlled (repressed) by the action of BTS in shoots and root stele, by targeting bHLH IVc transcription factors for degradation (indicated by a solid line inhibition symbol), and possibly also bHLH121/URI (dashed line inhibition symbol). In the root epidermis and cortex, BTSL2 targets FIT for degradation, and BTSL1 potentially targets IVc bHLH transcription factors. The overall effect of BTS and BTSL activity is to repress genes such as *IRT1*, *FRO2*, and *ZIF1*, preventing accumulation of Fe, as well as Zn. FBP inhibits FIT-dependent induction of *NAS1/2/4* in the root stele. Under Zn excess conditions (Fe/Zn ratio of ~0.05), Fe deficiency response genes are up-regulated, in particular *IMA* peptide genes and group Ib transcription factors. Specific IMA peptides inhibit the interaction between BTS and IVc bHLHs by competing for the ubiquitination substrate proteins, and a similar mechanism may interfere with BTSL-mediated turnover of bHLH121/URI, IVc bHLH, and FIT. Subsequently, *IRT1* and *FRO2* are up-regulated, allowing increased uptake of Fe, but additionally of Zn as well. Elevation of the cytosolic Zn concentration is known to inhibit IRT1 activity as it is internalized and removed, but this cannot prevent excessive Zn influx. Increased expression of the genes *NAS* and *ZIF1* enhances vacuolar sequestration of Zn–NA in root vacuoles, whilst promoting Fe transport to shoots. In the *btsl1 btsl2* mutant under Zn excess (not shown), the expression of FIT-dependent genes *IRT1* and *FRO2* and group Ib transcription factors remains high in the roots; however, the transcriptional response of Fe-regulated genes in the shoots is similar to the wild type under standard conditions, correlating with attenuated *IMA* expression (see text for details).

Analysis of mutants that are tolerant to external Zn, such as the *btsl1x2* mutant, could help answer outstanding questions in Zn and Fe crosstalk. Specifically, how are Zn and Fe signals integrated and by which regulators and regulatory mechanisms? Previously suggested points of crosstalk and regulation, which are not mutually exclusive, are the Zn-induced internalization of IRT1; competition of Fe and Zn for protein-binding sites; and disruption of systemic signalling mechanisms (Hanikenne *et al*., 2021). Our phenotypic, ionomic and transcriptomic analyses of the *btsl1x2* mutant in comparison with the wild type suggest a model to explain why excess Zn causes systemic Fe deficiency (Fig. 6) and how mutation of certain genes, in particular genes negatively affecting FIT activity (Table 1), suppress this phenomenon.

In the presence of excess Zn, wild-type Arabidopsis seedlings take up large amounts of Zn and this is translocated to the shoots. Thus, IRT1 internalization cannot prevent this large influx of Zn. Increased expression of genes involved in vacuolar Zn sequestration (*MTP3* and *ZIF1*, see Fig. 3C) indicates that part of the internalized Zn is rendered non-toxic by storing it in vacuoles. However, impaired root growth (Fig. 1D) suggests that not all excess Zn can be made harmless. Zn excess in the medium also leads to massive Fe accumulation in the roots (Fig. 2B) but not to increased Fe in the shoots. Based on the transcriptomics data, it appears that induction of the Fe deficiency response is likely to boost Fe acquisition by the roots, but that Fe cannot progress to the shoots. Our data are consistent with a possible deposition of Fe in the root apoplast, conceivably as a result of suboptimal FCR activity (Fig. 4), or in other locations where Fe cannot be removed by EDTA washes. For example, Quintana *et al.* (2022) observed Fe accumulation in/around the cell walls of cortex cells in an *irt1* mutant using Perls’ staining, and this pool of Fe was also resistant to EDTA washes. Because neither ferritin nor vacuolar Fe transporters are transcriptionally induced in response to excess Zn in wild-type roots (Fig. 3C), this apoplastic deposition seems the most likely scenario, which could be verified by detailed microscopy studies of cellular Fe pools in the roots in the future.

Lack of BTSL1 and BTSL2 partially prevents Fe accumulation in Zn-exposed roots, which is counterintuitive given that Fe uptake genes are more highly induced than in the wild type and only slightly more Fe is translocated to the shoots. Possibly, slightly increased Fe uptake initially suppresses Zn-induced internalization of IRT1 (Barberon *et al.*, 2014; Dubeaux *et al.*, 2018; Martin-Barranco *et al.*, 2020), allowing sufficient Fe uptake and subsequent transport to shoots, which switches off the shoot-to-root iron deficiency signalling. However, increased Fe uptake via IRT1 alone is not sufficient to confer Zn tolerance, and in fact the *idf1* mutant which is impaired in IRT1 turnover shows Zn accumulation and sensitivity to Zn excess (Dubeaux *et al.*, 2018). Interestingly, similar to *btsl1x2*, the *bts-1* mutant also showed elevated expression of Fe uptake genes under Zn excess, but the latter accumulates more Fe in both roots and shoots than the wild type. One possible explanation for the different metal accumulation patterns is that *BTS* is expressed in leaves as well as in the root stele (Selote *et al.*, 2015). Up-regulation of the Fe deficiency response in both shoots and roots is expected to generate increased capacity for Fe uptake and transport to shoots. To further explore this hypothesis, transcriptomics datasets of separated roots and shoots of *bts-1* would be required, to tease apart the expression patterns in whole seedlings reported by Zhu *et al.* (2022). In contrast, *btsl1x2* shows up-regulation of the root Fe deficiency response only (Fig. 3), and a small increase in shoot Fe under standard growth conditions (Hindt *et al.*, 2017) (Fig. 2B).

In addition to the striking difference in root Fe accumulation between the wild type and the *btsl* double mutant, another key difference is the physiological Fe status of the shoots based on transcriptomics data (Fig. 3). Wild-type shoots under Zn excess have only a slight decrease in Fe (Fig. 2B) but are chlorotic (Fig. 1B) and have high expression of genes normally induced by Fe deficiency (Fig. 3; Supplementary Fig. S8). In contrast, the *btsl1x2* double mutant has near-normal chlorophyll levels and is Fe replete according to transcriptomics analysis. This difference is not affected by exposing part of the root system to Zn excess (Fig. 5).

Interestingly, Tabata *et al*. (2022) observed an up-regulation of *IRT1* in wild-type root halves exposed to Fe sufficiency when the other root half was exposed to Fe deficiency, whereas we find no significant difference in *IRT1* expression in these conditions (Supplementary Fig. 10D). However, Tabata *et al.* (2022) exposed seedlings to test conditions for 24 h whilst we grew seedlings under these conditions for 7 d. The longer exposure of our seedlings might have resulted in differences that are less dramatic or lost entirely as Fe is transported from the sufficient root half to the deficient root half. Our results for the *btsl1x2* mutant are similar to those of Tabata *et al.* (2022) for the *ima7x* mutant (e.g. Fig. 4B) in which *IRT1* is more strongly up-regulated in the –Fe (not significant) or ++Fe (significant) exposed root halves. This finding might result from loss of systemic signalling in *ima7x* and insensitivity to IMA-regulated systemic signals in *btsl1x2.*

Taken together, our results suggest that wild-type plants exposed to Zn excess may become trapped in a feed-forward loop of impaired Fe uptake, resulting in physiological Fe limitation in shoots and subsequent systemic Fe deficiency signalling to roots (Fig. 6). This feed-forward cycle observed in the wild type is similar to that seen in the *frd3* mutant, which shows constitutive activation of the root Fe deficiency response due to shoot-borne deficiency signalling, despite overaccumulating Fe in roots (Scheepers *et al.*, 2020). This cycle can be partially broken at the level of FIT through mutations that lead to increased FIT protein levels and activity, as in *btsl1x2* (this study) and *fbp* (Chen *et al.*, 2018), or mutations in upstream regulators such as the *bts* mutant (Zhu *et al.*, 2022). It would be interesting to investigate whether natural variation in *BTSL* or *BTS* expression patterns, or ability to interact with transcription factor targets (FIT, Ivc, or IVb) or IMA proteins, impacts Fe and Zn phenotypes.

In addition, we propose that the tight regulation of FIT by BTSL1/2 is a trade-off between preventing Fe toxicity against preventing Zn toxicity under fluctuating nutrient availability. BTSL1/2 have a physiologically important role in rapidly removing FIT and switching off Fe uptake when roots are newly exposed to Fe after a period of deficiency (Rodriguez-Celma *et al.*, 2019). This mechanism prevents a large flux of Fe into the plant that would be toxic. When BTSL1 and BTSL2 are absent, slightly enhanced Fe uptake is beneficial to outcompete with Zn for uptake and at protein-binding sites, and the Fe deficiency response triggered by Zn excess is suppressed. Thus, not having BTSL proteins, which are specific to dicotyledon plants, helps plants cope better with excess Zn in the environment, but it would be disadvantageous when Fe is in excess. It would be interesting to investigate natural genetic variation on soils with contrasting Fe and Zn concentrations, and see if there are accession that have lost functional BTSL proteins.

In summary, by regulating FIT and also FIT-independent transcriptional networks, the BTSL proteins are part of an important control point in balancing Fe and Zn uptake and preventing toxicity.

## Supplementary data

The following supplementary data are available at *JXB* online.

Table S1. qRT–PCR primers used in the study.

Dataset S1. RNA-seq data of a select Fe-responsive gene set significantly differentially expressed in response to Zn excess.

Dataset S2. RNA-seq data of the full list of genes significantly differentially expressed in response to Zn excess.

Fig. S1. The *btsl1 btsl2* phenotypic response to Zn excess.

Fig. S2. The *btsl1 btsl2* phenotypic response to Mn deficiency and excess.

Fig. S3. Principal component analysis (PCA) and comparison of the number of differentially expressed genes in the shoot transcriptomic response to Zn excess for the wild type and *btsl1 btsl2*.

Fig. S4. Gene Ontology (GO) analysis and comparison of number of differentially expressed genes in the root transcriptomic response to Zn excess for the wild type and *btsl1 btsl2*.

Fig. S5. Hierarchical clustering of differentially expressed genes in wild-type and *btsl1 btsl2* roots in response to Zn excess

Fig.S6. Gene Ontology (GO) analysis of differentially expressed genes in wild-type and *btsl1x2* shoots in response to Zn excess.

Fig. S7. qRT–PCR verification of Fe deficiency-responsive genes in roots.

Fig. S8. Hierarchical clustering of differentially expressed genes in wild type and *btsl1 btsl2* shoots in response to Zn excess.

Fig. S9. Shoot phenotype and shoot expression of *bHLH39* from the split.root experiment.

Fig. S10. Root expression of *IMA1*, *FER1*, *bHLH38*, and *IRT1* from the split-root experiment.

erad243_suppl_supplementary_figures_S1-S10_table_S1

erad243_suppl_supplementary_dataset_S1

erad243_suppl_supplementary_dataset_S2

## Data Availability

The data supporting the findings of this study are available within the supplementary data online or from the corresponding author, Janneke Balk, upon request.

## Abbreviations

bHLH: basic helix–loop–helix
BTS: BRUTUS
BTSL: BRUTUS-LIKE
FBP: FIT-binding protein
FCR: ferric chelate reductase
Fe: iron
FIT: FER-like iron deficiency-induced transcription factor
FRO2: ferric reduction oxidase 2
GSH: glutathione
HMA: heavy metal ATPase
IRT1: iron-regulated transporter1
Mn: manganese
MTP: metal tolerance protein
NA: nicotianamine
NAS: nicotianamine synthase
ZIF: zinc-induced facilitator
Zn: zinc
