# Supplementary material for: BRUTUS-LIKE (BTSL) E3 ligase-mediated fine-tuning of Fe regulation negatively affects Zn tolerance of Arabidopsis
Source: J Exp Bot. 2023 Jul 2;74(18):5767–82. doi: 10.1093/jxb/erad243 (PMC10540732; doi:10.1093/jxb/erad243)
Supplement: erad243_suppl_supplementary_figures_S1-S10_table_S1 [file erad243_suppl_supplementary_figures_s1-s10_table_s1.pdf]

Supplementary Table 1: qRT-PCR primers

Gene name	Primer name	Sequence (5' – 3')	Source
ZIF1	*ZIF1* F	TCTGGGGAATTGTGGCTGAT	C Stanton
	ZIF1 R	GGCCTTCATTGTTCCAAGCA	
FRO3	*FRO3* F	CCCGGCGATTAGAAGGAGAT	C Stanton
	FRO3 R	GTTCCGCGACTGGAGAAATC	
ACTIN2	*ACTIN2* F	GATGAGGCAGGTCCAGGAATC	Czechowski *et al.*, 2005
	ACTIN2 R	GTTTGTCACACACAAGTGCAT	
IRT1	*IRT1* F	CACCATTCGGAATAGCGTTAGG	Rodriguez-Celma *et al.*, 2019
	IRT1 R	CCAGCGGAGCATGCATTTA	
FRO2	*FRO2* F	TTACCCGATCGACCACAACAC	Rodriguez-Celma *et al.*, 2019
	FRO2 R	CCGCACTACAAGTCGCCATTAT	
bHLH38	*bHLH38* F	TTTCACAAACTTCGGTTGGCC	Rodriguez-Celma *et al.*, 2019
	bHLH38 R	CTGACGAAACAGATACTCCCAAGCT	
bHLH39	*bHLH39* F	GGCCATCAACGGGAGAGTAC	Rodriguez-Celma *et al.*, 2019
	bHLH39 R	GCTCCATAAGTCTCCTCCGG	
IMA1	*IMA1* F	TGATTGTAATTTAGGAGGAAACAAAA	Grillet *et al.*, 2018
	IMA1 R	TCAATCCACAAGTAAACATCTATGG	
FER1	*FER1* F	TCCCCAGTTAGCTGATTTCG	Grillet *et al.*, 2018
	FER1 R	CTTTGCCGATCATCCTTAGC	
TIP41	*TIP41* F	GTGAAAACTGTTGGAGAGAAGCAA	Grillet *et al.*, 2018
	TIP41 R	TCAACTGGATACCCTTTCGCA	

References

Czechowski T, Stitt M, Altmann T, Udvardi MK, Scheible WR. 2005. Genome-wide identification and testing of superior reference genes for transcript normalization in Arabidopsis. Plant Physiol. **139**, 5-17

Grillet L, Lan P, Li W, Mokkapati G, Schmidt W. 2018. IRON MAN is a ubiquitous family of peptides that control iron transport in plants. Nature Plants **4**, 953-963.

Rodriguez-Celma J, Connorton JM, Kruse I, Green RT, Franceschetti M, Chen YT, Cui Y, Ling HQ, Yeh KC, Balk J. 2019. *Arabidopsis* BRUTUS-LIKE E3 ligases negatively regulate iron uptake by targeting transcription factor FIT for recycling. Proceedings of the National Academy of Sciences, USA **116**, 17584-17591.

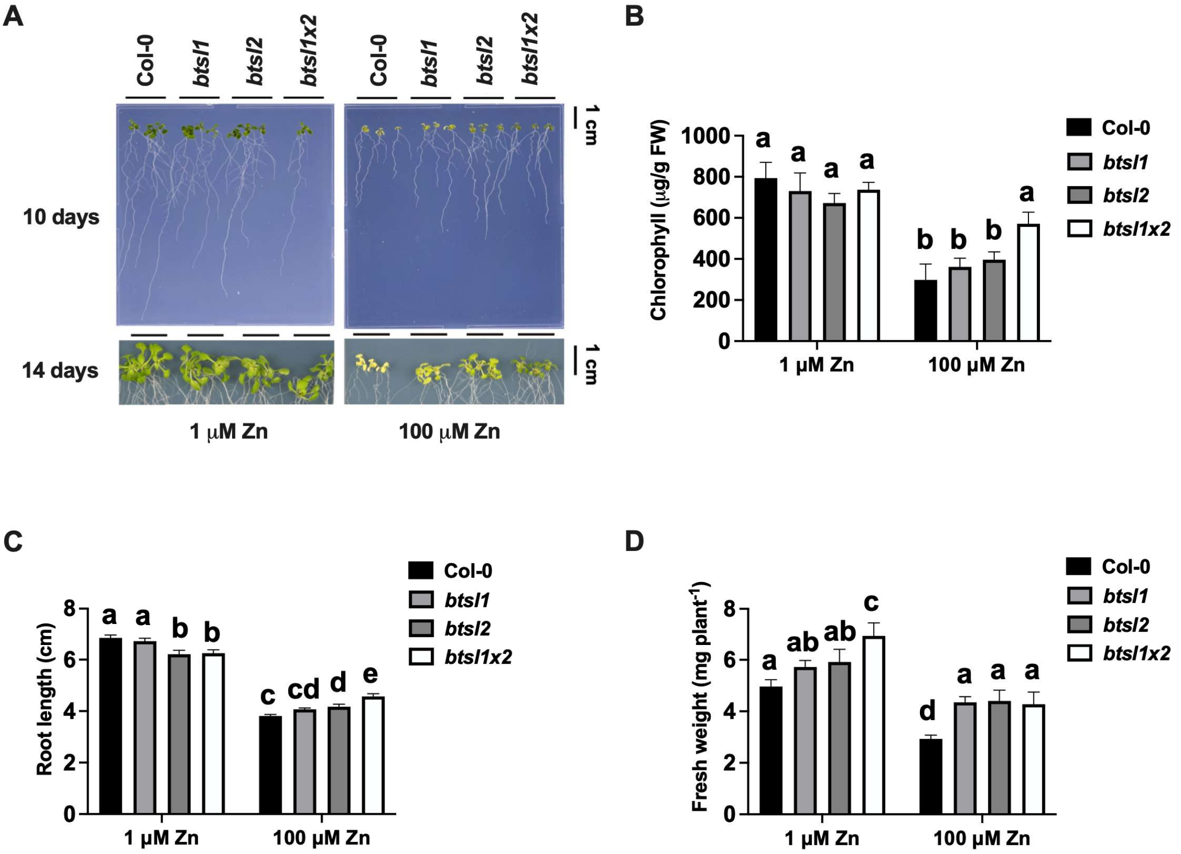

Figure S1: The *btsl1 btsl2* double mutant displays Zn tolerance

A) Representative photographs of 14-day-old seedlings of wild type (Col-0) and *btsl1 btsl2* double mutant (*btsl1x2*), grown on standard medium (1 µM $ZnSO_4$, 5 µM Fe(HBED)) or Zn excess (100 µM $ZnSO_4$, 5 µM Fe(HBED))

B) Total chlorophyll normalized to fresh weight (FW) in shoots from seedlings in (A).

C) Primary root length of 10-day-old seedlings.

D) Shoot biomass, in mg fresh weight (FW), of seedlings in (A).

For B – D, data represent mean values (± SEM) from three independent experiments, each comprising six plants per genotype and condition. Statistically significant differences are indicated by letters ($p < 0.05$) as determined by two-way ANOVA followed by Tukey HSD post-hoc test.

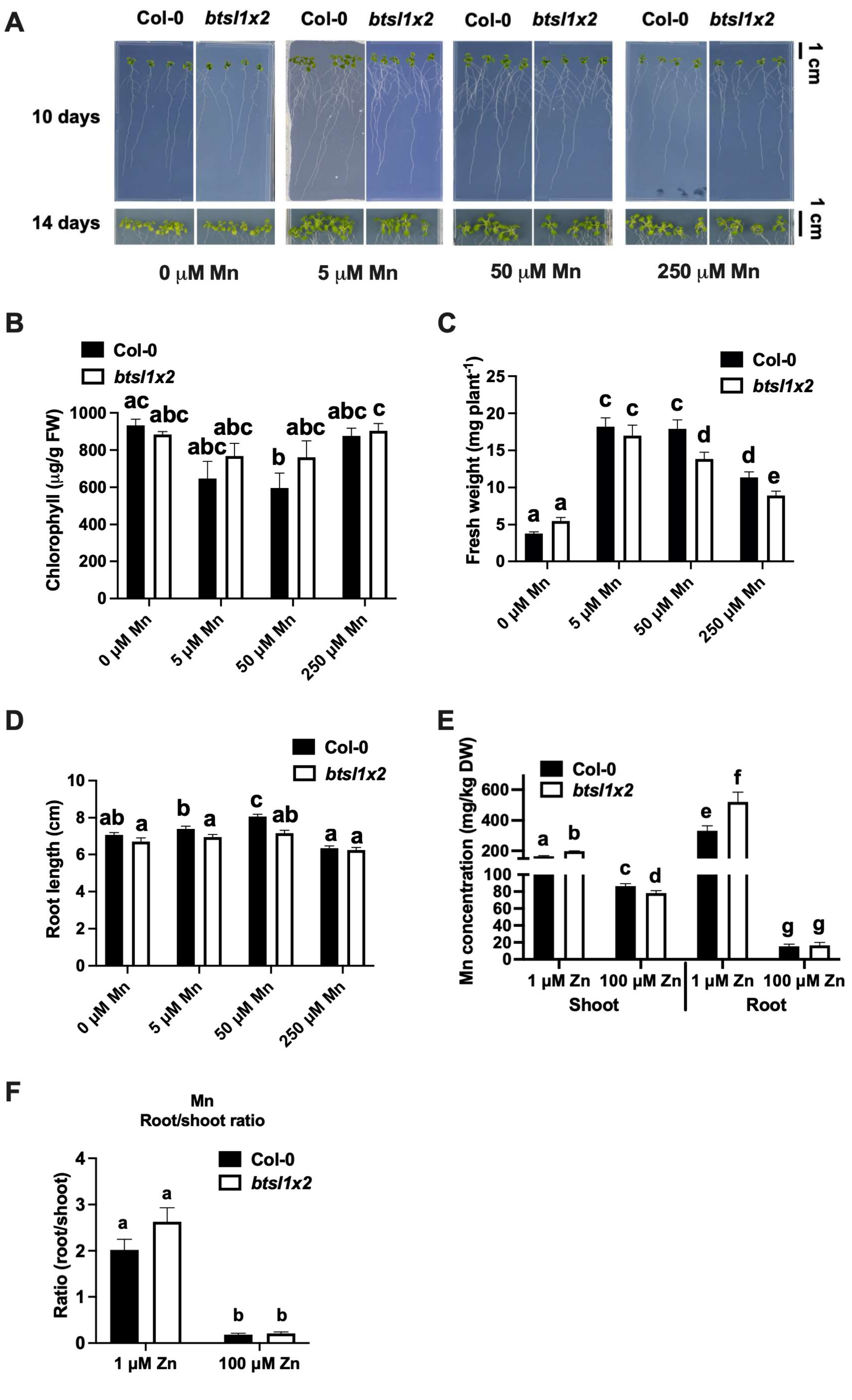

Figure S2: The *btsl1 btsl2* double mutant is relatively more tolerant to Mn deficiency

A) Representative photographs of 14-day-old seedlings of wild type (Col-0) and *btsl1 btsl2* double mutant (*btsl1x2*), grown on modified Hoagland medium with 1 µM ZnSO4 and 5 µM Fe(HBED) (standard) in the presence of 0 µM, 5 µM, 50 µM or 250 µM $MnSO_4$ as indicated. (Continued on next page.)

Figure S2 (continued from previous page)
B) Total chlorophyll normalized to fresh weight (FW) in shoots from seedlings in (A).
C) Shoot biomass, in mg fresh weight (FW), of seedlings in (A).
D) Primary root length of 10-day-old seedlings.
E) The concentration of Mn in the roots and shoots of 14-d-old seedlings of wild type (Col-0) and *btsl1x2* grown on standard (1 µM $ZnSO_4$, 5 µM Fe(HBED)) or Zn excess (100 µM $ZnSO_4$, 5 µM Fe(HBED)). Roots were desorbed with chelators to remove apoplastic cations prior to element analysis.
F) Zn-to-Fe ratio of shoots and roots. For B-E data represent mean values (± SEM) from three independent experiments, each comprising ten plants per genotype and condition. Statistically significant differences are indicated by letter ($p < 0.05$) as determined by two-way ANOVA followed by Tukey HSD post-hoc test.

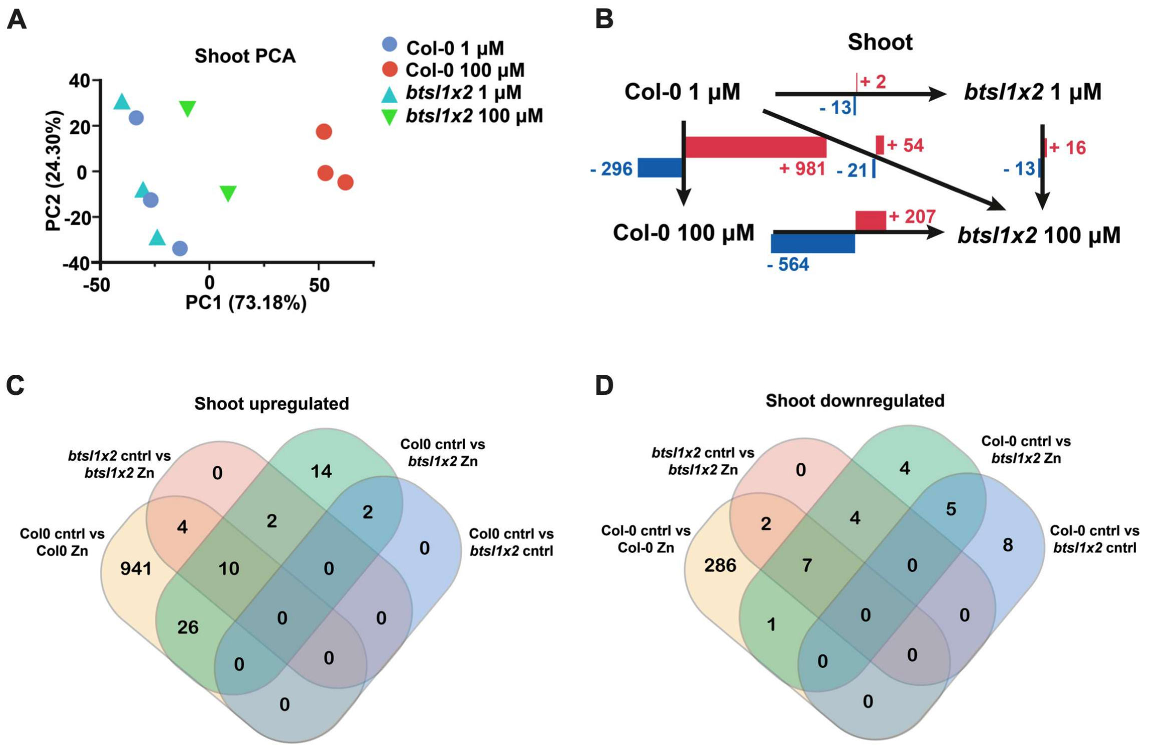

Figure S3: The *btsl1 btsl2* double mutant has little shoot transcriptomic response to Zn excess

A) Principal component analysis (PCA) of shoot sample transcripts PC1 = principle component 1, PC2 = principle component 2, (%) is the percentage of variance explained by each PC.

B) Number of significantly (adjusted p-value > 0.05, $\log_2$(fold change) ≥ 1) upregulated (+) or downregulated (-) genes between all pairwise comparisons.

C) upregulated and D) downregulated DEGs in four pairwise comparisons of interest (Col-0 1 µM Zn vs *btsl1x2* 1 µM Zn; Col-0 1 µM Zn vs Col-0 100 µM Zn; Col-0 1 µM Zn vs *btsl1x2* 100 µM Zn; *btsl1x2* 1 µM Zn vs *btsl1x2* 100 µM Zn) and the overlap between these gene sets.

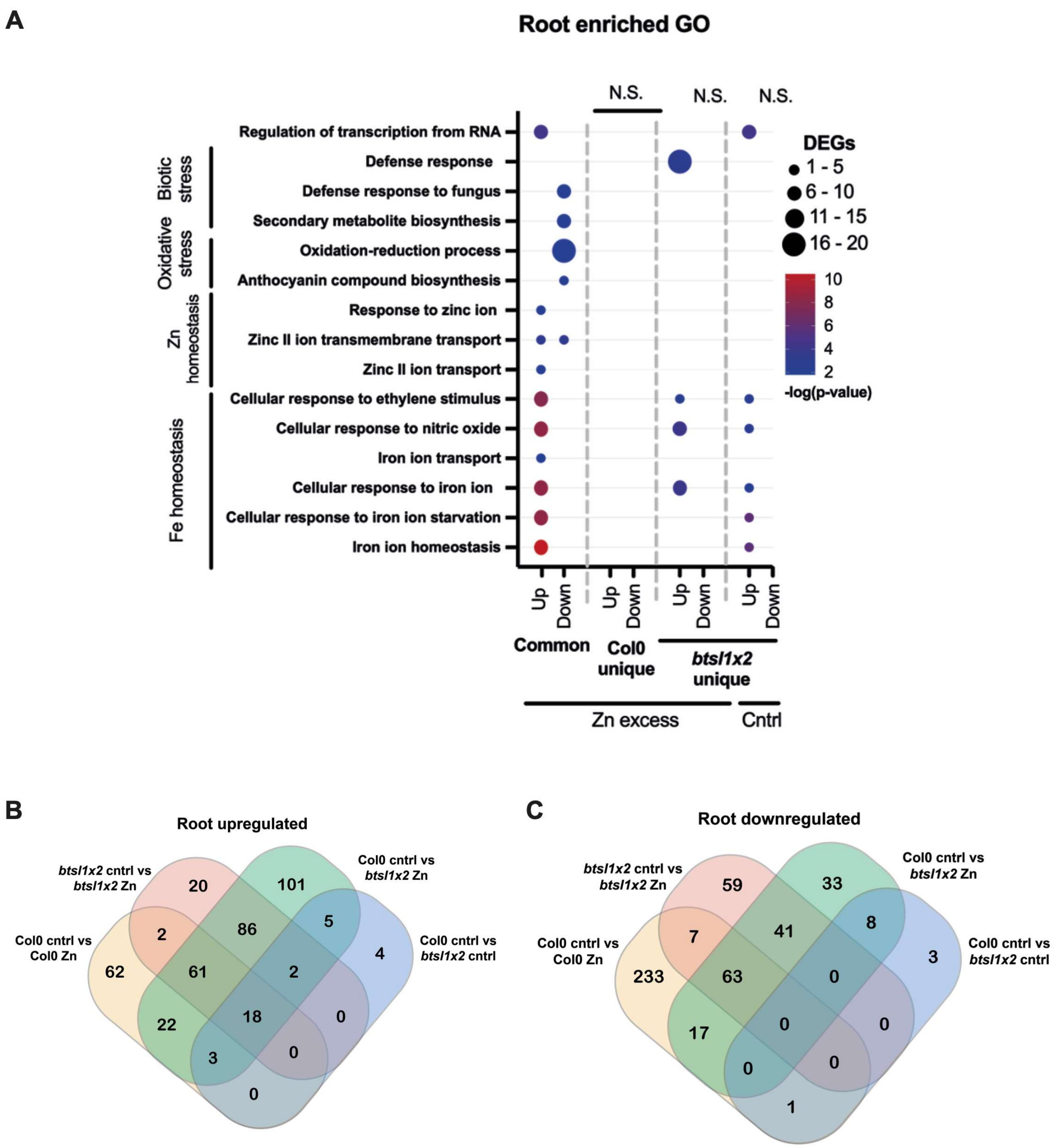

Figure S4: Fe deficiency-responsive genes are constitutively overexpressed in *btsl1 btsl2* double mutant roots. A) Gene Ontology (GO) enrichment analysis of common and unique Zn-responsive DEGs in Col-0 and *btsl1x2* roots. All significant (padj < 0.05) terms are shown on the vertical axis and grouped by similar function. The size of each point represents the number of DEGs associated with each term and the colour shows –log(p-value). C) upregulated and D) downregulated DEGs in four pairwise comparisons of interest (Col-0 1 µM Zn vs btsl1x2 1 µM Zn; Col-0 1 µM Zn vs Col-0 100 µM Zn; Col-0 1 µM Zn vs btsl1x2 100 µM Zn; btsl1x2 1 µM Zn vs btsl1x2 100 µM Zn) and the overlap between these gene sets.

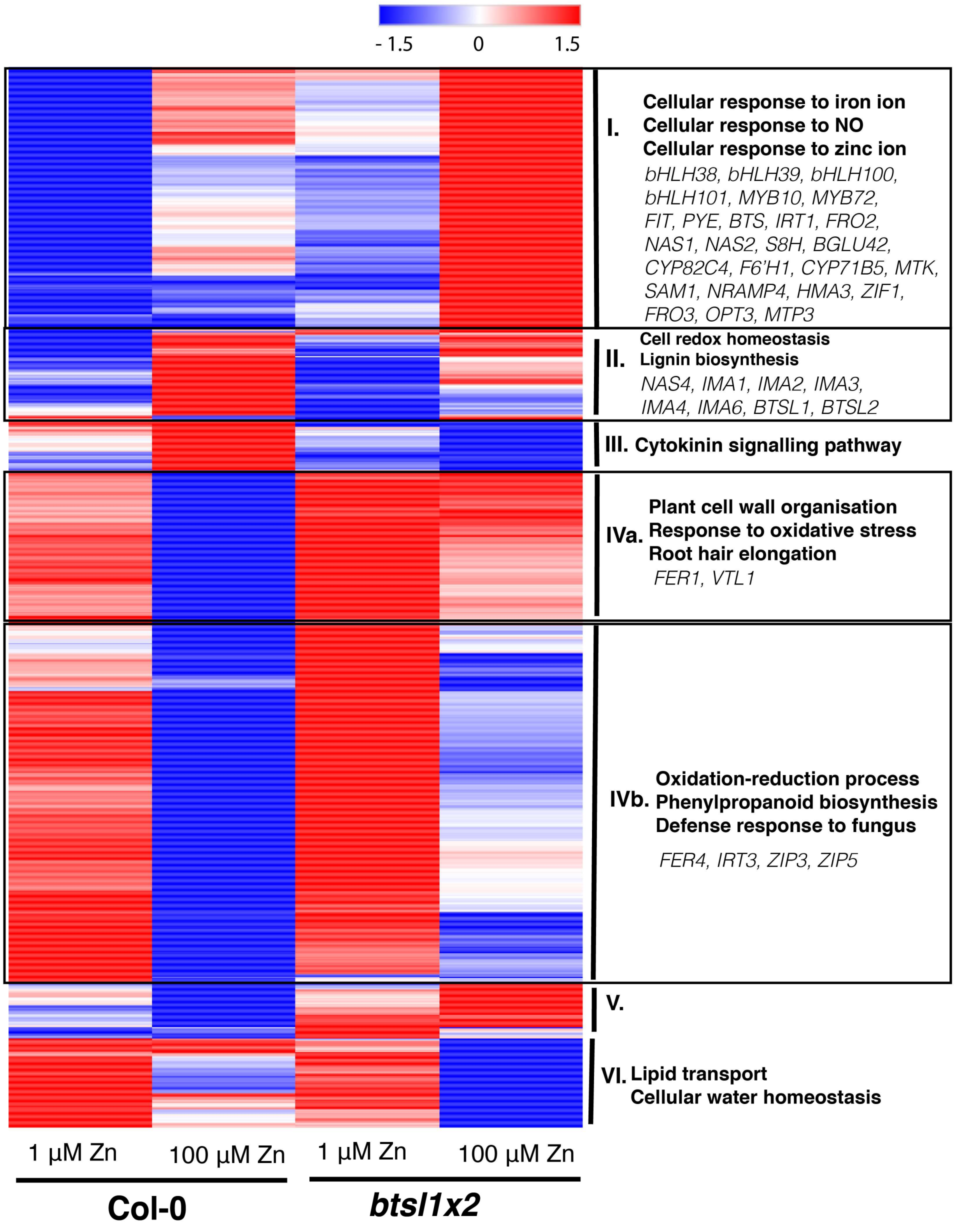

Figure S5: Hierarchical clustering of root DEGs

Major clusters are number I-VI and annotated for significantly (padj < 0.05) enriched GO terms and Fe/Zn homeostasis genes. Expression is represented as standard deviation from the mean.

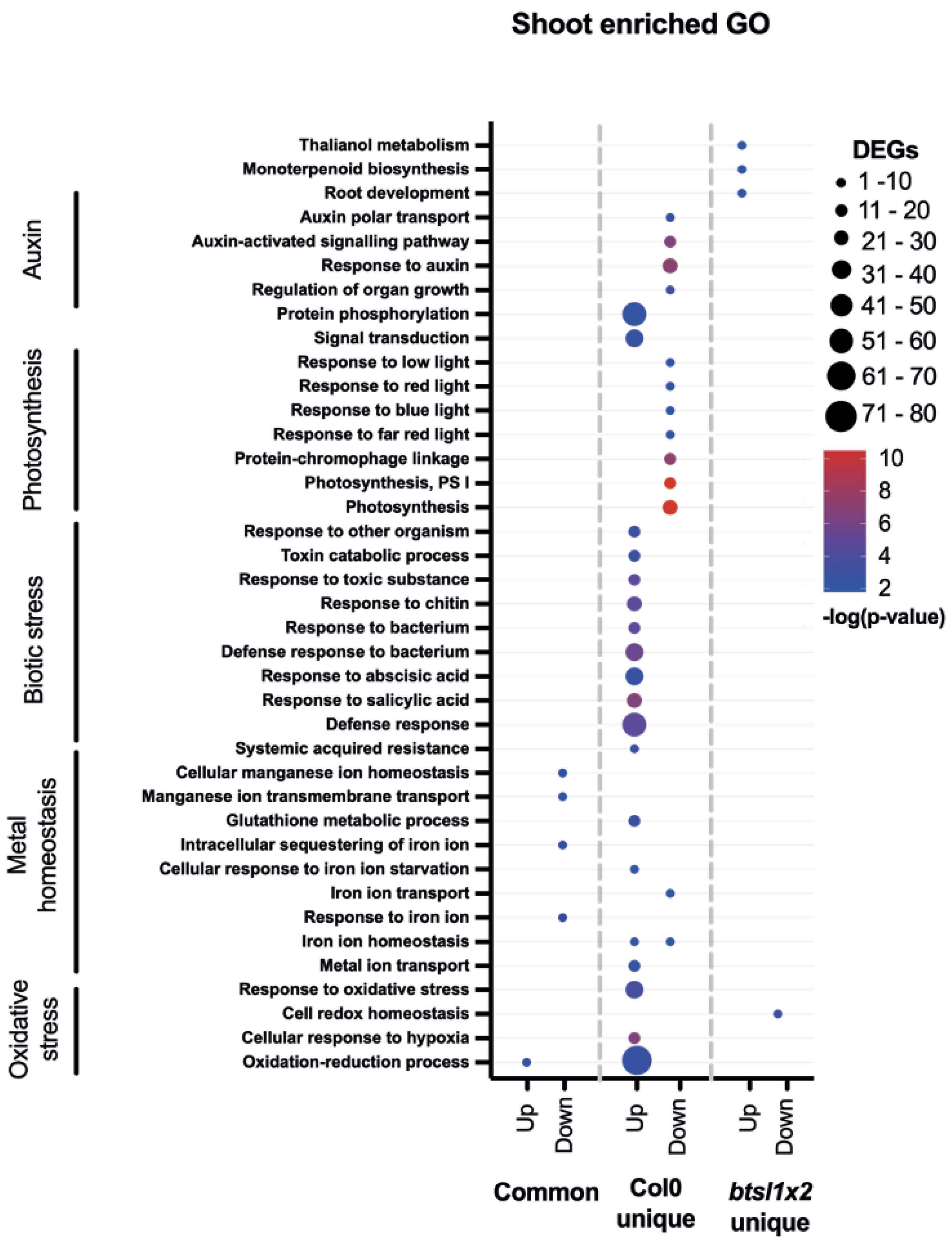

Figure S6: Fe deficiency-responsive genes are not differentially expressed in *btsl* double mutant shoots under Zn excess

Gene Ontology (GO) enrichment analysis of common and unique Zn-responsive DEGs in Col-0 and *btsl1x2* shoots. All significant (padj < 0.05) terms are shown on the vertical axis and grouped by similar function. The size of each point represents the number of DEGs associated with each term and the color shows –log(p-value).

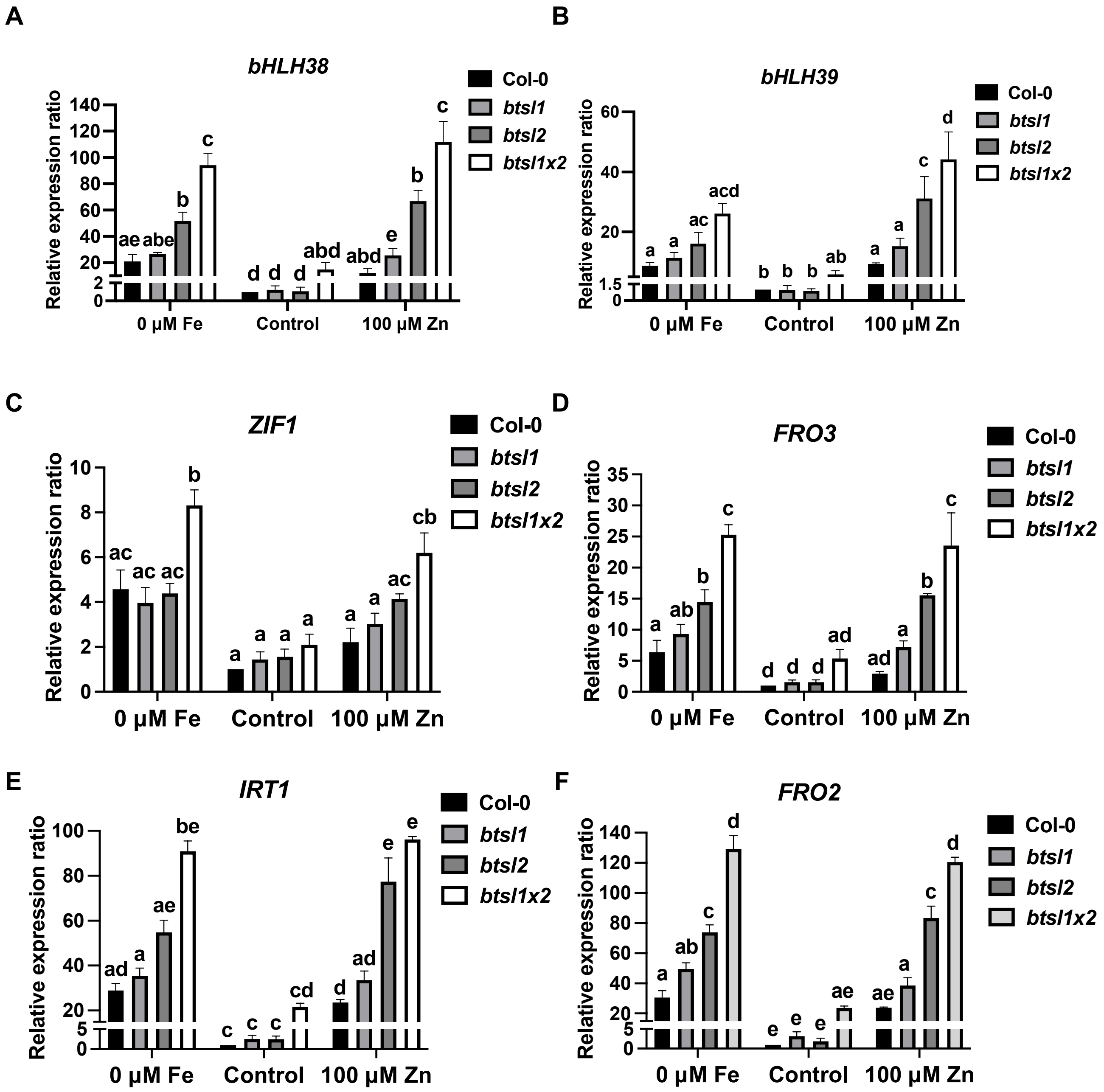

Figure S7: qRT-PCR verification of select Fe deficiency-responsive genes in *btsl* single and double mutant roots under Zn excess and Fe deficiency growth conditions.

The expression of A) *bHLH38;* B) *bHLH39;* C) *FRO3* D) *ZIF1,* E) *IRT1* and F) *FRO2* in Col-0, *btsl1*, *btsl2* and *btsl1x2* 14-d-old seedlings grown continuously on Fe deficiency (1 µM $ZnSO_4$, 0 µM Fe(HBED)), Zn excess (100 µM $ZnSO_4$, 5 µM Fe(HBED)) or control (1 µM $ZnSO_4$, 5 µM Fe(HBED)) agar plates. Fold change is relative to Col-0 control (expression level set as "1") and normalised to reference genes *ACTIN2* and *TIP41*. Data represent mean values (± SEM) from three independent experiments, each comprising five plants per genotype and condition, and each repeated as three technical replicates. Statistically significant differences are indicated by letters ($p < 0.05$) as determined by two-way ANOVA followed by Tukey HSD post-hoc test.

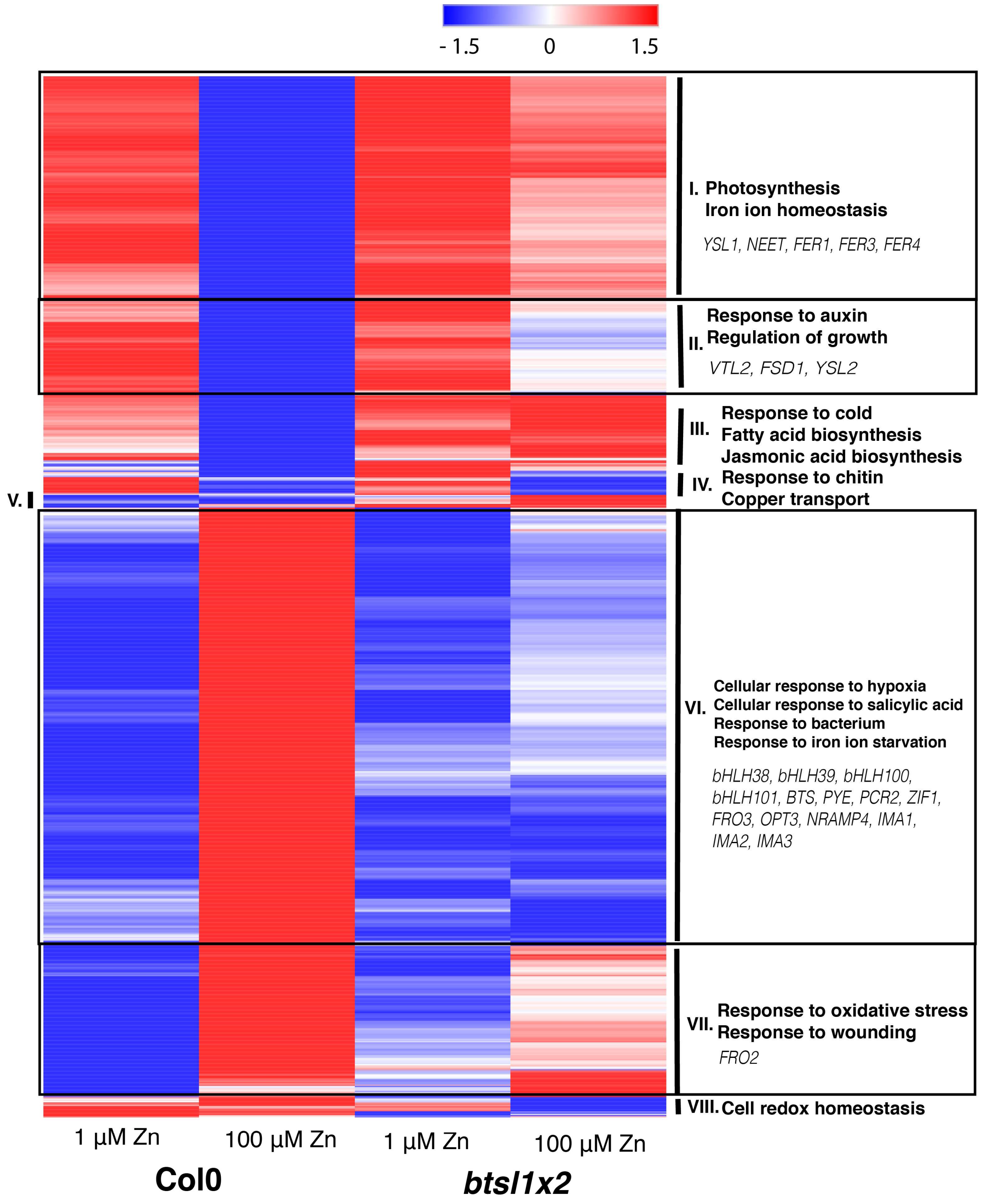

Figure S8: Hierarchical clustering of shoot DEGs
Major clusters are number I-VI and annotated for significantly (padj < 0.05) enriched GO terms and Fe/Zn homeostasis genes. Expression is represented as standard deviation from the mean.

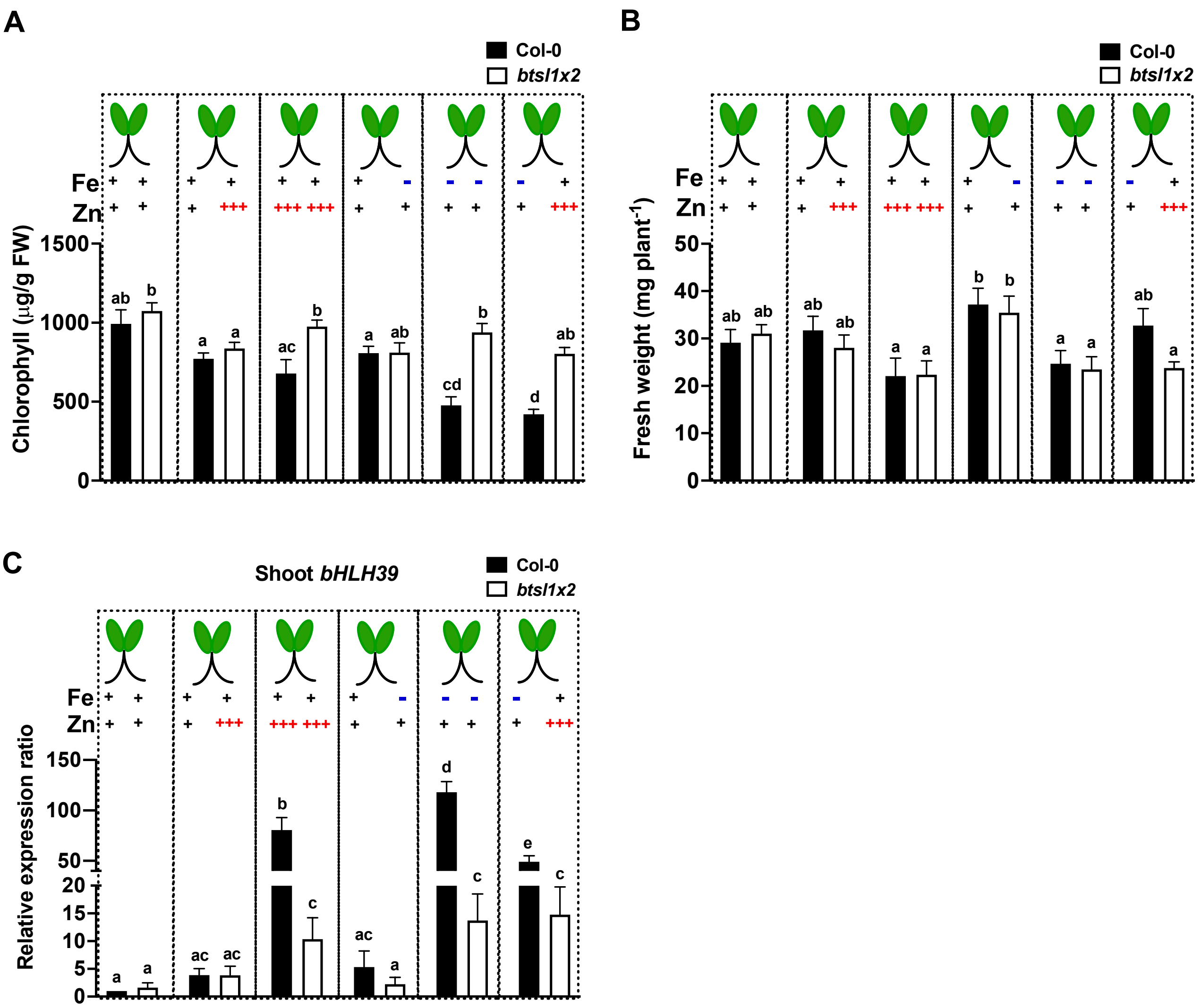

Supplementary Figure 9: Systemic Fe signalling in *btsl1 btsl2* mutant seedlings in response to Fe deficiency and Zn excess

Wild type (Col-0) and *btsl1 btsl2* mutant (*btsl1x2*) seedlings were germinated on standard medium (1 µM $ZnSO_4$, 5 µM Fe(HBED)). The primary root was excised at day 5 to promote development of lateral roots. At 10 days, seedlings were transferred to split agar plates with the indicated Fe and Zn concentrations. Samples for RNA extraction were taken 7 days later.

A) Shoot fresh weight (FW)

B) Chlorophyll content

C) Expression of *bHLH38* in shoots in response to Zn excess and Fe deficiency exposure in different halves of the roots system.

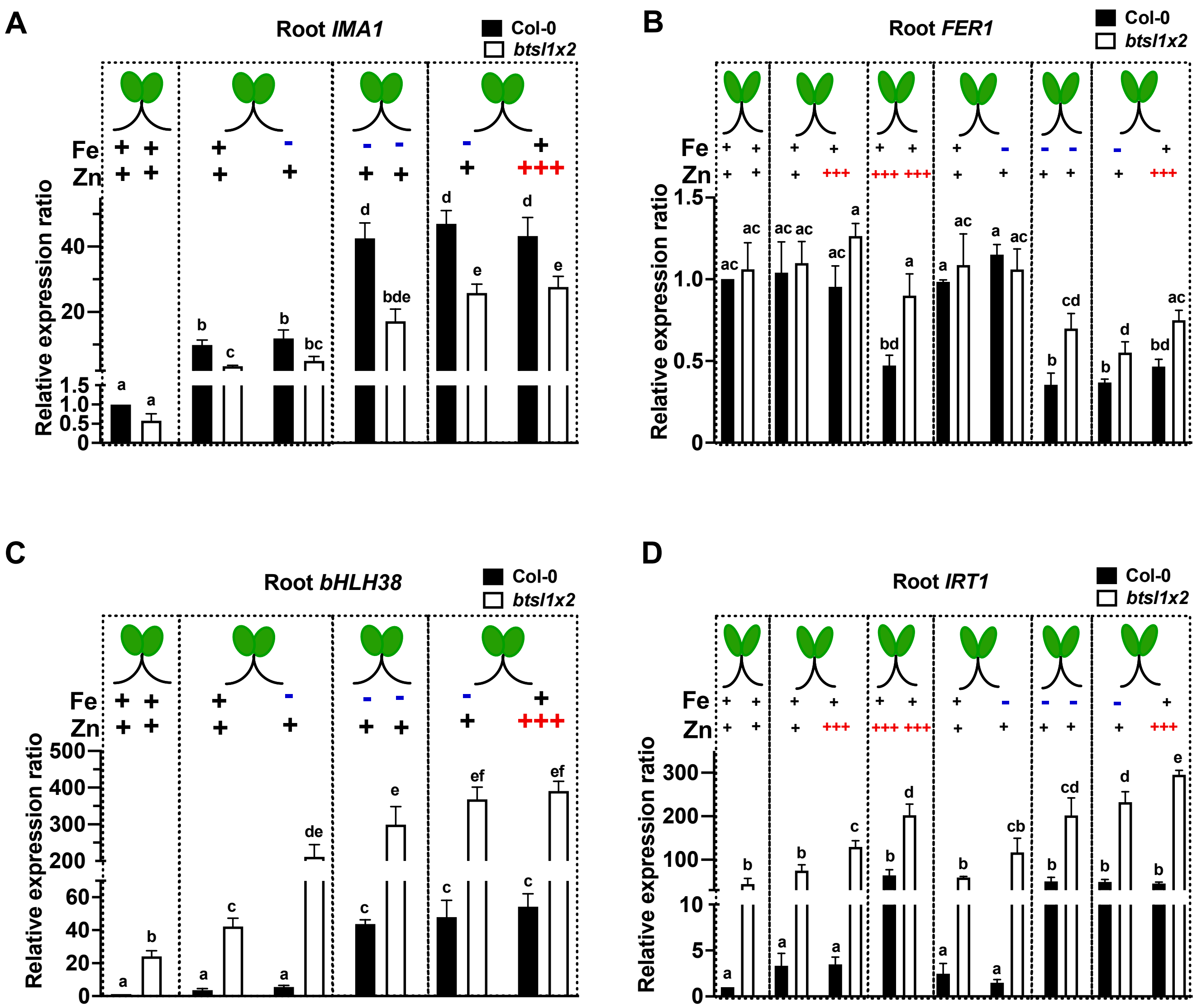

Supplementary Figure 10: Systemic Fe signalling in *btsl1 btsl2* mutant seedlings in response to Fe deficiency and Zn excess, expression of select Fe deficiency genes in roots

Wild type (Col-0) and *btsl1 btsl2* mutant (*btsl1x2*) seedlings were germinated on standard medium (1 µM $ZnSO_4$, 5 µM Fe(HBED)). The primary root was excised at day 5 to promote development of lateral roots. At 10 days, seedlings were transferred to split agar plates with the indicated Fe and Zn concentrations. Samples for RNA extraction were taken 7 days later.

A) Expression of *IMA1,* B) *FER1*, C) *bHLH38* and D) *IRT1* in roots in response to Zn excess and Fe deficiency exposure in different halves of the roots system.
